# Healthcare spending in U.S. emergency departments by health condition, 2006–2016

**DOI:** 10.1371/journal.pone.0258182

**Published:** 2021-10-27

**Authors:** Kirstin Woody Scott, Angela Liu, Carina Chen, Alexander S. Kaldjian, Amber K. Sabbatini, Herbert C. Duber, Joseph L. Dieleman

**Affiliations:** 1 Department of Emergency Medicine, University of Michigan, Ann Arbor, MI, United States of America; 2 Institute for Health Metrics and Evaluation, University of Washington, Seattle, WA, United States of America; 3 Harvard Medical School, Boston, MA, United States of America; 4 Department of Emergency Medicine, University of Washington, Seattle, WA, United States of America; University of Malta Faculty of Health Sciences, MALTA

## Abstract

**Background:**

Healthcare spending in the emergency department (ED) setting has received intense focus from policymakers in the United States (U.S.). Relatively few studies have systematically evaluated ED spending over time or disaggregated ED spending by policy-relevant groups, including health condition, age, sex, and payer to inform these discussions. This study’s objective is to estimate ED spending trends in the U.S. from 2006 to 2016, by age, sex, payer, and across 154 health conditions and assess ED spending per visit over time.

**Methods and findings:**

This observational study utilized the National Emergency Department Sample, a nationally representative sample of hospital-based ED visits in the U.S. to measure healthcare spending for ED care. All spending estimates were adjusted for inflation and presented in 2016 U.S. Dollars. Overall ED spending was $79.2 billion (CI, $79.2 billion-$79.2 billion) in 2006 and grew to $136.6 billion (CI, $136.6 billion-$136.6 billion) in 2016, representing a population-adjusted annualized rate of change of 4.4% (CI, 4.4%-4.5%) as compared to total healthcare spending (1.4% [CI, 1.4%-1.4%]) during that same ten-year period. The percentage of U.S. health spending attributable to the ED has increased from 3.9% (CI, 3.9%-3.9%) in 2006 to 5.0% (CI, 5.0%-5.0%) in 2016. Nearly equal parts of ED spending in 2016 was paid by private payers (49.3% [CI, 49.3%-49.3%]) and public payers (46.9% [CI, 46.9%-46.9%]), with the remainder attributable to out-of-pocket spending (3.9% [CI, 3.9%-3.9%]). In terms of key groups, the majority of ED spending was allocated among females (versus males) and treat-and-release patients (versus those hospitalized); those between age 20–44 accounted for a plurality of ED spending. Road injuries, falls, and urinary diseases witnessed the highest levels of ED spending, accounting for 14.1% (CI, 13.1%-15.1%) of total ED spending in 2016. ED spending per visit also increased over time from $660.0 (CI, $655.1-$665.2) in 2006 to $943.2 (CI, $934.3-$951.6) in 2016, or at an annualized rate of 3.4% (CI, 3.3%-3.4%).

**Conclusions:**

Though ED spending accounts for a relatively small portion of total health system spending in the U.S., ED spending is sizable and growing. Understanding which diseases are driving this spending is helpful for informing value-based reforms that can impact overall health care costs.

## Introduction

The emergency department (ED) fills a vital role in the health system, caring for patients with acute medical illness and injury 24 hours a day, 7 days a week, and serving as a critical safety net to millions of Americans each year [1, 2]. At the same time, ED visits can be expensive, particularly when compared to alternative sites of care [3]. As such, the ED has been subject to policymaker scrutiny as momentum has grown to curb healthcare spending and payers have moved towards delivery reforms and alternative payment models designed to improve the value of healthcare services more broadly [4]. Efforts to promote improved value around emergency care have tended to focus on preventing avoidable ED visits that can be treated in less costly settings, as well as those that reduce low-value testing and interventions [5–8].

Beyond policymaker and payer interest in decreasing ED spending, patients also have concerns about the cost of ED care. Recent media coverage and ongoing legislative debates have brought greater attention to “surprise billing” practices and high ED charges, including during the current COVID-19 pandemic [9–12]. Despite this heightened attention to ED costs, there are surprisingly few studies that provide a comprehensive assessment of ED spending over time, including how ED spending varies across health conditions and patient populations. Prior work has generally shown overall ED spending to be a relatively small contributor to national health spending as compared to other sectors, but has lacked detail on where these dollars have been allocated, has been limited to specific conditions, or focused only on a subset of ED patients [13–17]. These varying approaches make it difficult to assess ED spending trends over time and across key groups, which would be helpful as policymakers seek to improve its value.

In this context, the current study aims to describe ED spending in the U.S. across a range of policy relevant variables from 2006 to 2016. First, the distribution of ED spending is summarized across age groups, patient sex, payer, and 154 health conditions from 2006 to 2016. Second, ED spending is delineated by disposition (“treat-and-release” ED visits versus those resulting in hospitalization). Third, ED spending per visit estimates are summarized over time and across health conditions.

## Methods

### Conceptual framework

This paper complements a recent study published by the Disease Expenditure (DEX) project at the Institute for Health Metrics and Evaluation that provides health spending estimates across seven settings of care from 1996–2016, including the ED [18]. In this current study, a more detailed analysis of ED estimates is presented, including ED spending trends by age, sex, condition, payer, disposition, and per visit from 2006 to 2016.

### Data

Spending estimates presented in this analysis rely on an existing dataset constructed for the broader DEX study, which leveraged 198 source-years of microdata to estimate health spending across all health care services, including the ED [18]. The DEX ED spending estimates from 2006 through 2016 rely primarily on ED visit data obtained from the Nationwide Emergency Department Sample (NEDS) [19]. As detailed in that methodology, DEX ED spending estimates are based on a random selection of 50% of the NEDS data (S1 and S2 Tables in S1 Appendix). Since 2014 NEDS data were not available to that research team at the time of the analysis, an average of 2013 and 2015 ED spending was used for 2014. Additional datasets used for generating the DEX ED estimates used in this analysis include the 2006–2016 Medical Expenditure Panel Survey, which captures information about ED payments and charges using a nationally representative household survey; Nationwide Inpatient Sample, which captures comorbidities and inpatient spending; and National Health Expenditure Accounts, which captures total national healthcare spending.

### Estimating ED spending by year, age, sex, payer, and health condition

The primary outcomes of interest were ED spending by health condition, age, sex, payer, and year (2006–2016), which come from a DEX dataset that has been described in greater detail elsewhere [18]. The following summarizes key portions in the development of the DEX spending estimates focused on the ED. First, the NEDS facility charge for ED services, as well as age, sex, and diagnosis codes were extracted for each visit. Each visit and charge (which accounted for inflation and transformed into 2016 U.S. Dollars ($) across all years) was assigned one of 38 unique age-sex groups as well as 154 health conditions, which map to 15 aggregated health categories (S3 and S4 Tables in S1 Appendix). All DEX spending estimates, including those in the ED, underwent a comorbidity adjustment (described in detail elsewhere [20]), that accounts for how comorbidities (e.g., tobacco use) may affect spending associated with the primary diagnosis and reassigns charges accordingly [17, 18, 20]. Second, facility charges were used to estimate total spending after adjusting for each health condition, age, and year combination using data from the Medical Expenditure Panel Survey, which reports both charges and payments for facilities and physicians [21]. The final spending estimates shown in this analysis thus aim to reflect ED payments (and not charges) for both facility and physician services in the ED. Third, Medical Expenditure Panel Survey data were used to model a ratio that was applied to national payer distribution estimates as provided by the National Health Expenditure Accounts, and made it possible to disaggregate ED spending into three payer groups: public (Medicare, Medicaid, or other public insurance programs), private, or out-of-pocket (deductibles, copays, or other payments not covered by insurance) [22]. Fourth, a hierarchical model was used to ensure estimates are not driven by extreme outliers, drawing information across age and time as needed. Fifth, following prior work [18], disaggregated spending estimates were scaled by proportionally adjusting spending by health condition, age, and sex, so that disaggregated ED spending summed by year was equal to the total ED spending determined by the National Health Expenditure Accounts (S1 Appendix).

### ED disposition analysis

ED spending estimates were further disaggregated by disposition into two groups, which are exhaustive of all NEDS visits: (i) visits whose care was managed by the ED (“treat-and-release” visits), and (ii) visits which resulted in an inpatient admission. The fraction of total ED spending that was attributable to the “treat-and-release” group was calculated using NEDS visit-level data. This fraction was applied to the total ED spending estimates to yield estimates for the “treat-and-release” group by age, sex, health condition, payer, and year.

### ED spending per visit analysis

Each NEDS visit from the processed dataset was counted, and information was captured to assign that visit to the appropriate age-sex group, health condition, and year. This study did not generate a volume estimate by payer. Finally, ED spending per visit was calculated by dividing the total spending by the total number of visits by year. A sub-analysis was conducted to illustrate how ED spending per visit varies by health condition.

### Reporting

The estimates reported herein are the mean of the bootstrapped sample of 1000 draws from each NEDS visit used in the analytic sample and are provided with associated confidence intervals (CIs) at the 2.5th and 97.5th percentiles. Each of the 1000 draws is carried through the entirety of the estimation process. Spending changes are summarized in both absolute terms (difference between 2016 and 2006) and relative changes (creating a population-standardized annualized rate of change (AROC), which accounts for changes in the population (age, sex, and size) from 2006 and 2016). All estimates were adjusted for inflation and reported in 2016 U.S. dollars. All analyses were completed using Stata (StataCorp), version 13.1; R (R Foundation), version 3.5.1; and Python (Python Software Foundation), version 3.6.

Research related to the DEX project underwent review and received approval from the Institutional Review Board at the University of Washington.

## Results

### ED spending trends across time

**Fig 1:**
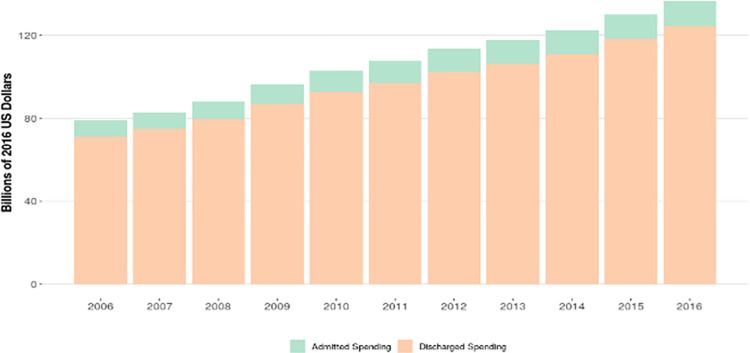
Total emergency department spending per year by disposition, 2006–2016.

**Fig 1** shows that total ED spending was $79.2 billion (CI, $79.2 billion-$79.2 billion) in 2006 and grew to $136.6 billion (CI, $136.6 billion-$136.6 billion) in 2016. ED spending accounted for 3.9% (CI, 3.9%-3.9%) of total healthcare spending in the U.S. in 2006 and this grew to 5.0% [CI, 5.0%-5.0%] by 2016 (S1 Fig in S1 Appendix). The population-adjusted annualized growth rate for ED spending between 2006 and 2016 was 4.4% (CI, 4.4%-4.5%), compared to the whole healthcare sector, which grew at 1.4% (CI, 1.4%-1.4%) over that same time period (S5 Table in S1 Appendix).

### ED spending by payer

The payer distribution for ED spending shifted minimally between 2006 and 2016. Public insurance accounted for 47.8% (CI, 47.8%-47.8%) in 2006 and 46.9% (CI, 46.9%-46.9%) or $64.0 billion (CI, $64.0 billion-$64.0 billion) in 2016. Private insurance accounted for 47.7% (CI, 47.7%-47.7%) in 2006, which grew to 49.3% (CI, 49.3%-49.3%) of all ED spending or $67.3 billion (CI, $67.3 billion-$67.3 billion) in 2016 (S2 Fig in S1 Appendix). Out-of-pocket ED spending accounted for 4.9% (CI, 4.9%-4.9%) (or $3.5 billion [CI, $3.5 billion-$3.5 billion]) of all ED spending in 2006, which fell to 3.9% (CI, 3.9%-3.9%) (or $5.3 billion [CI, $5.3 billion-$5.3 billion]) in 2016. After adjusting for changes in inflation, population size, and age, ED spending by private payers witnessed the greatest relative increase in spending at 5.2% (CI, 5.2%-5.2%), followed by public payers at 3.8% (CI, 3.7%-3.8%), and out-of-pocket payments at 3.4% (CI, 3.4%-3.4%) (S6 Table in S1 Appendix).

### ED spending by health condition

**Fig 2:**
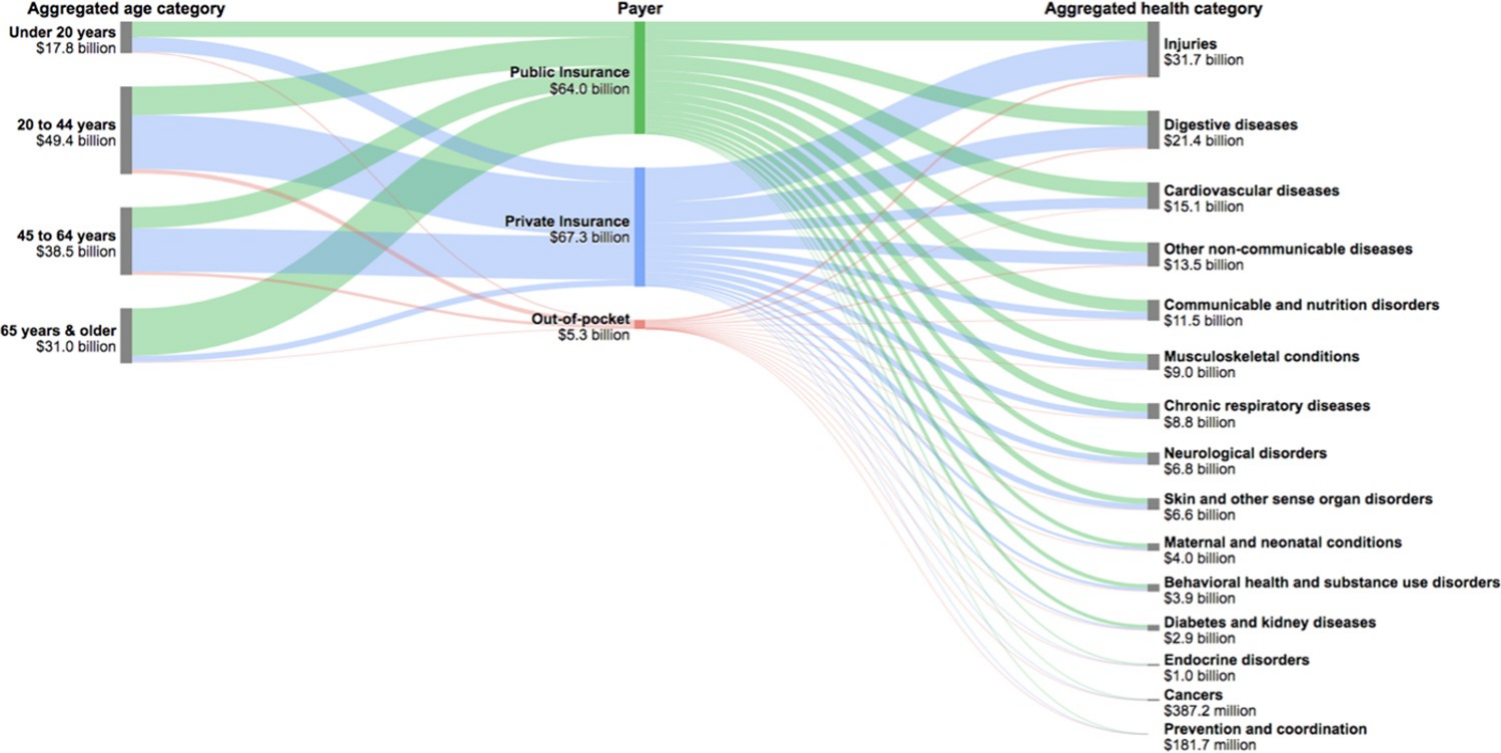
Emergency department spending across aggregated age groups, payer, and aggregated health categories, 2016 ($ billions).

**Fig 2** summarizes ED spending across 15 aggregated health categories, payer, and age groups for 2016. Injuries as a category comprised the highest modeled ED spending estimated at $31.7 billion (CI, $31.3 billion-$33.2 billion) in 2016, followed by digestive diseases ($21.4 billion [CI, $19.7 billion-$23.0 billion]) and then cardiovascular diseases ($15.1 billion [CI, $14.0 billion-$16.3 billion]). This order was the same in 2006 (results not shown). Spending in each of the 15 broader health categories is further disaggregated into the 154 individual health conditions (S4 Table in S1 Appendix). Injuries—the aggregated health category accounting for the highest modeled ED spending—is made up of fourteen granular conditions, with four of these appearing in the 25 highest-ranking ED spending conditions as shown in Table 1: road injuries (ranked 1^st^), falls (ranked 3^rd^), unintentional injuries due to mechanical forces (ranked 7^th^), and other unintentional injuries (ranked 13^th^).

**Table 1:** Changes in overall Emergency Department (ED) spending and ED spending per visit over time for the 25 health conditions with the most ED spending in 2016.

	ED Spending						ED Spending Per Visit		
	Total Spending 2016		Absolute Change (2006–2016)		Relative Change (2006–2016)		2006	2016	Relative Change
	$ Billions	Rank	$ Billions	Rank	AROC (%)	Rank	$/visit	$/visit	AROC (%)
**All Health Conditions**	**136.61**	**n/a**	**57.38**	**n/a**	**4.4**	**n/a**	**660.05**	**943.16**	**3.4**
Road injuries	9.88	1	4.24	2	4.8	45	954.28	1606.98	5.1
Urinary diseases	9.41	2	4.39	1	5.2	44	959.73	1289.57	2.8
Falls	8.46	3	2.87	4	3.0	71	613.57	856.53	3.2
Other digestive diseases	5.47	4	2.30	8	4.2	56	809.02	1071.44	2.6
Low back and neck pain	5.18	5	2.49	5	5.6	39	657.95	1001.82	4.0
Skin and subcutaneous diseases	5.01	6	2.31	7	5.3	42	389.83	575.99	3.7
Unintentional injuries due to mechanical forces	4.76	7	0.79	26	1.1	87	472.91	701.63	3.9
Ischemic heart disease	4.59	8	1.48	13	1.9	84	1349.54	1714.86	2.4
Inflammatory bowel disease	4.57	9	2.37	6	6.6	30	798.72	1245.93	4.3
Hypertension	4.44	10	1.87	9	3.8	58	1128.53	1238.20	0.8
Gallbladder and biliary diseases	4.16	11	3.02	3	12.4	3	1029.37	1808.60	5.7
Lower respiratory tract infections	4.12	12	1.30	16	2.9	73	490.36	661.70	3.0
Other unintentional injuries	3.55	13	1.45	14	4.5	52	475.53	699.38	3.7
Asthma	3.52	14	1.45	15	4.7	47	631.61	910.39	3.6
Chronic obstructive pulmonary disease	3.31	15	1.71	10	5.2	43	952.20	1286.51	3.1
Other musculoskeletal conditions, including joint pain	3.29	16	1.63	11	5.8	36	600.79	770.85	2.3
Gynecological diseases	3.16	17	1.28	17	4.7	49	825.62	1119.68	3.0
Appendicitis	3.07	18	0.68	28	2.0	82	1220.23	2630.62	7.9
Other neurological conditions	2.97	19	1.53	12	6.2	32	712.85	991.60	3.1
Upper respiratory tract infections	2.73	20	0.86	23	3.4	64	314.41	398.78	2.2
Diabetes mellitus	2.34	21	1.07	19	4.7	48	739.71	929.56	2.3
Interpersonal violence	2.09	22	0.67	29	3.4	65	708.71	1117.61	4.5
Migraine	1.95	23	1.03	20	7.2	24	591.80	1123.03	6.5
Other chronic respiratory diseases	1.92	24	1.14	18	8.3	17	576.50	991.70	5.3
Cerebrovascular disease	1.72	25	0.59	31	2.2	80	1047.16	1395.76	3.0

*Note*: Health conditions are sorted by those with the highest levels of ED spending as of 2016 (see S8 Table for complete list in S1 Appendix). Absolute change = the difference in total ED spending between 2016 and 2006. Rank columns permit for how health conditions compare with one another in terms of absolute levels or changes in spending. The relative change measure is a population-standardized Annualized Rate of Change (AROC), which accounts for changes in the population between 2006 and 2016. It provides a relative estimate of the change in spending growth (or spending per visit growth) noted from 2006 and 2016 for all conditions, and then by each individual health condition. All estimates are reported in 2016 USD ($).

**Fig 3:**
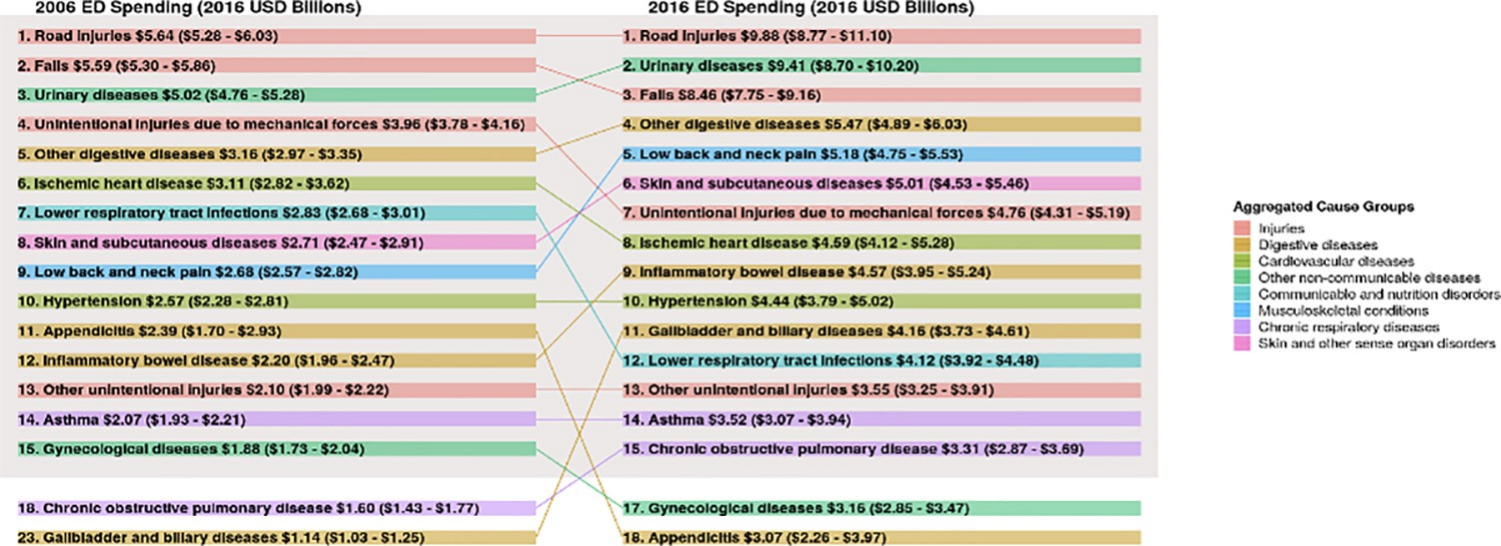
Arrow diagram summarizing the 15 individual health conditions with highest estimated emergency department spending levels in 2006 and 2016. *Note*: Health conditions are ranked by estimated ED spending in both 2006 and 2016 (both using 2016 USD) and placement in the ranking is illustrated in the arrow diagram. Each of the 154 health conditions captured by the Disease Expenditure Project (DEX) map to 1 of 15 aggregated cause groups (or referred to as 15 aggregated health categories), which are shown in the legend and displayed in Fig 1. Of note, ED spending estimates were mapped to only 150 of the 154 DEX conditions, as four health conditions (larynx cancer, well newborn care, well dental, and well person) either did not appear in the ED data or were systematically excluded as a primary diagnosis.

**Fig 3** illustrates the 15 highest-ranking individual health conditions in terms of modeled ED spending and how this has changed over time. Road injuries accounted for the most ED spending over time, increasing from $5.6 billion (CI, $5.3 billion-$6.0 billion) in 2006 to $9.9 billion (CI, $8.8 billion-$11.1 billion) in 2016. Falls increased from $5.6 billion (CI, $5.3 billion-$5.9 billion) in 2006 to $8.5 billion (CI, $7.7 billion-$9.2 billion) in 2016. Spending on urinary diseases (e.g., urinary tract infection and other urologic emergencies or concerns) surpassed spending on falls in 2016 at $9.4 billion (CI, $8.7 billion-$10.2 billion), increasing from $5.0 billion (CI, $4.8 billion-$5.3 billion) in 2006. Taken together, these three conditions accounted for 14.1% (CI, 13.1%-15.1%) of all ED spending in 2016.

**Table 1** summarizes both absolute and relative changes in ED spending over time for the 25 health conditions with the most ED spending in 2016, which account for 77.4% (CI, 76.5%-78.1%) of all ED spending that year. For absolute changes in spending between 2006 and 2016 among the 154 health conditions, urinary diseases, road injuries, and gallbladder and biliary diseases saw the largest absolute changes in ED spending, increasing by $4.4 billion (CI, $3.8 billion-$5.0 billion), $4.2 billion (CI, $3.3 billion-$5.3 billion), and $3.0 billion (CI, $2.6 billion-$3.5 billion) from 2006 to 2016, respectively.

The relative change in spending, measured by the annualized growth rate, across all health conditions was 4.4% (CI, 4.4%-4.5%). Among the 154 health conditions with at least $1 billion in ED spending in 2016, the three conditions with the greatest relative changes in spending from 2006 to 2016 were gallbladder and biliary diseases (12.4% [CI, 11.1%-13.8%]), septicemia (12.1% [CI, 10.1%-13.5%]), and cirrhosis of the liver (10.7% [CI, 8.8%-12.4%]). Among this list, gallbladder and biliary diseases, saw some of the highest levels of change in both absolute and relative growth, accounting for $4.2 billion [CI, $3.7 billion-$4.6 billion] of ED spending in 2016.

### ED spending by demographics

The distribution of spending by sex remained relatively stable over the entire study period, with women accounting for nearly 60% of estimated ED spending. Specifically, females accounted for a total of $44.8 billion (CI, $44.3 billion-$45.4 billion) (or 56.6% [CI, 56.0%-57.3%]) of ED spending in 2006 and then $79.6 billion ([CI, $78.7 billion-$80.5 billion], or 58.3% (CI, 57.6%-58.9%) in 2016.

Young adults (20–44 years) accounted for the largest proportion of overall ED spending across all years (S3 Fig in S1 Appendix). In 2006, the 20–44 age group accounted for 35.6% (CI, 35.0%-36.2%) of ED spending (or $28.2 billion [CI, $27.8 billion—$28.7 billion]) and a similar proportion of overall ED spending in 2016, 36.2% (CI, 35.6%-36.7%) (or $49.4 billion [CI, $48.6 billion—$50.2 billion]) (**Fig 2**).

ED spending by condition among these younger age-sex groups differ by magnitude and order. In 2016, the three conditions accounting for the most ED spending among females ages 20–44 were urinary diseases ($2.6 billion [CI, $2.4 billion-$2.9 billion]), gynecological diseases ($2.2 billion [CI, $2.0 billion-$2.4 billion]), and road injuries ($2.2 billion [CI, $1.9 billion-$2.5 billion]), whereas ED spending for males ages 20–44 went to road injuries ($2.3 billion [CI, $2.0 billion-$2.6 billion]), exposure to mechanical forces ($1.2 billion [CI, $1.1 billion-$1.4 billion]), and urinary diseases ($1.1 billion [CI, $1.0 billion-$1.3 billion]).

### ED spending by disposition

Of the $136.6 billion in total ED spending in 2016, $124.5 billion [CI, $124.1 billion-$124.8 billion] (or 91.2% [CI, 91.0%-91.5%]) was spent on the treat-and-release ED group (S4 Fig in S1 Appendix). This was similar to 2006 (90.0% [CI, 89.7%-90.2%]). The health conditions with the highest levels of ED spending among the treat-and-release group mirrored the top condition list for all ED spending: road injuries, urinary diseases, and falls; a total of $9.4 billion [CI, 8.3 billion-$10.6 billion], $8.9 billion [CI, $8.2 billion-$9.7 billion], and $7.8 billion [CI, $7.2 billion-$8.5 billion] was spent on the treat-and-release subgroup for these conditions, respectively.

The remaining balance of ED spending was attributed to the ED services rendered for those visits that resulted in a hospital admission. Among the hospitalized group, the conditions with the highest estimated level of ED spending in 2016 were septicemia ($957.7 million [CI, $802.7 million-$1.1 billion]), ischemic heart disease ($821.8 million [CI, $730.6 million-$952.0 million]), and falls ($622.0 million [CI, $554.7 million-$682.1 million]) (S7 Table in S1 Appendix).

### ED spending per visit over time and by health condition

**Fig 4:**
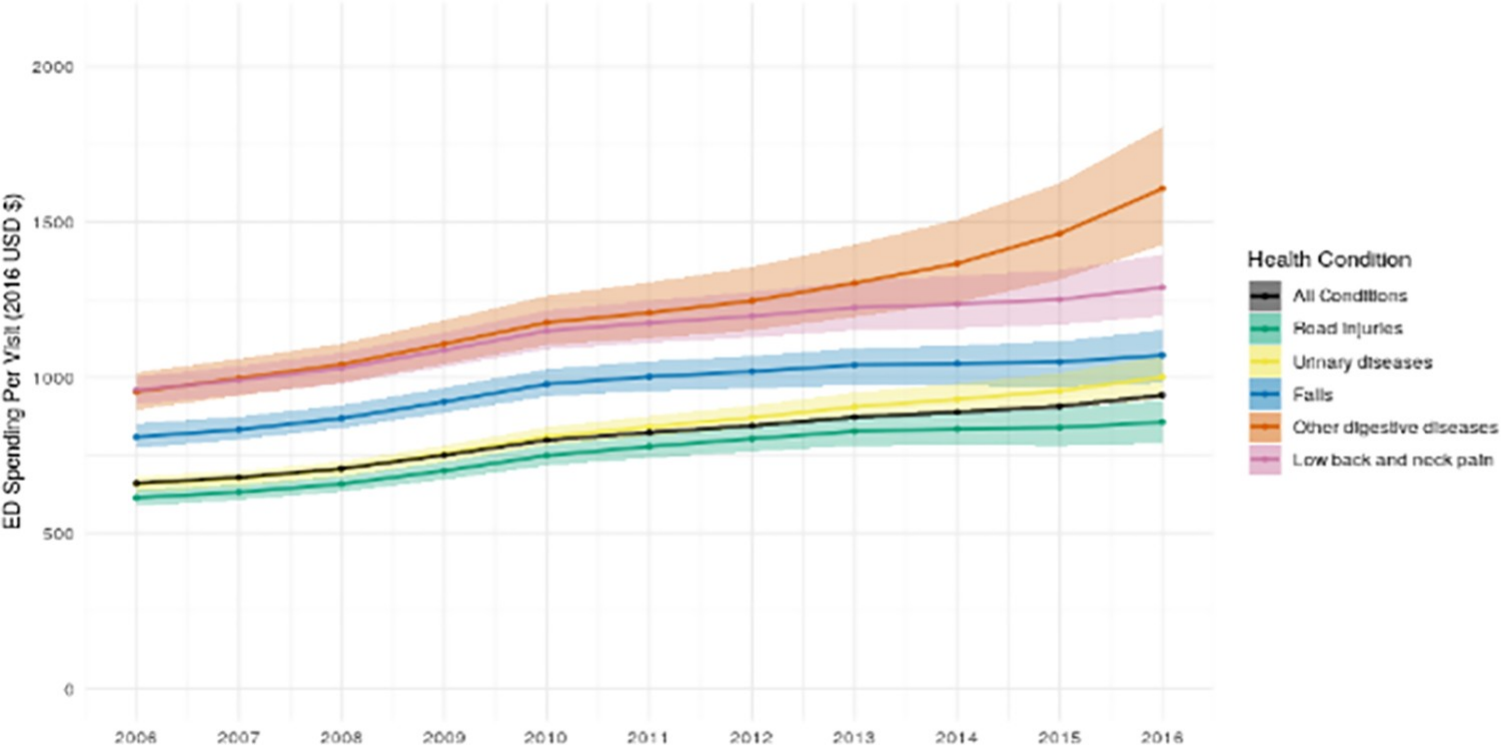
Emergency department spending per visit overall and for the 5 health conditions with the most ED spending in 2016.

In 2006, there were approximately 120.0 million (CI, 119.1 million-120.9 million) ED visits, increasing to 144.8 million (CI, 143.6 million-146.2 million) ED visits in 2016, reflecting a population-adjusted annualized growth rate of 1.04% (CI, 0.96%-1.11%) over this period (S5 Fig in S1 Appendix). Overall ED spending per visit grew from $660.0 (CI, $655.1-$665.2) in 2006 to $943.2 (CI, $934.3-$951.6) in 2016, or a population-adjusted annualized growth rate of 3.4% (CI, 3.3%-3.4%) (**Fig 4**).

**Fig 4** summarizes the five health conditions that accounted for the highest levels of estimated ED spending in 2016 (a total of 28.1% [CI, 27.1%-29.1%] of all ED spending in that year) and the variable increases in spending per visit over time across these conditions. For instance, ED spending per visit for road injuries was $954.3 (CI $896.3-$1016.0) in 2006 growing to $1607.0 (CI $1426.2-$1804.9) in 2016, representing an annualized growth rate of 5.1% (CI, 4.2%-6.1%). **Table 1** shows how ED spending per visit had a positive trajectory for all but one of the 25 conditions that had the highest levels of ED spending in 2016. The exception was treatment for hypertension, which was $1128.5 (CI, $1012.8-$1228.0) in 2006, increasing to $1301.9 (CI, $1169.6-$1449.9) by 2010, and then decreasing to $1238.2 (CI, $1063.4-$1399.5) by 2016 (S6 Fig in S1 Appendix).

## Discussion

This analysis demonstrates growth in both ED spending and visits between 2006 and 2016, totaling $136.6 billion (CI, $136.6 billion-$136.6 billion) and 144.8 million (CI, 143.6 million-146.2 million) visits in 2016. While ED care comprises only 5.0% (CI, 5.0%-5.0%) of the $2.71 trillion of national health spending in 2016 [18], ED spending grew on average 4.4% (CI, 4.4%-4.4%) per year since 2006, outpacing the healthcare sector, which grew at an annualized rate of 1.4% (CI, 1.4%-1.4%).

Prior evidence has consistently shown that the ED has historically accounted for a rather small proportion of overall national healthcare spending [13–15, 17]. However, the relative trajectory of spending and demand for ED care over the past decade has occurred in the setting of policymaker and payer enthusiasm to implement value-based reforms to reduce ED spending [3, 4], divert low-acuity ED visits to lower-cost alternatives including urgent care centers [23–25], emergence of high deductible health plans [26, 27], and upstream innovations such as primary care innovations to reduce what some would describe as preventable ED visits [6]. This growth may have persisted in spite of these pressures due a variety of reasons, including patient preference to seek ED care [28], outpatient provider reliance upon the ED for conducting intensive diagnostic workups for their patients [1, 29], increasing provision of ED services [30], and increasingly complex patient presentations [31, 32].

The majority of ED spending was paid in nearly equal parts between private and public payers between 2006 and 2016. However, growth was more prominent among private payers even despite major public insurance expansion efforts during this study period. Though prior work has also shown that private payers cover the majority of ED spending [33], additional research is needed to explore why these payer trends exist and the adequacy of compensation. This study, which found an overall lower absolute level of out-of-pocket ED spending compared to others [34], showed a relative decline in out-of-pocket ED spending over time. It will be important to monitor this trend in light of the recent slight uptick to the uninsured rate [35, 36], growth of high-deductible health plans [26, 27], and concern regarding out-of-network billing practices [11, 37].

Among the fifteen health categories that ED spending is delineated by in this study, injuries accounted for the highest ED spending over time. Four of the fourteen individual health conditions that make up this broader category of injuries ranked in the top twenty-five conditions in terms of modeled ED spending in 2016 (road injuries, falls, unintentional injuries due to mechanical forces, and other unintentional injuries). Among the 154 individual health conditions, road injuries, urinary diseases, and falls consistently accounted for the highest absolute levels of ED spending over time. Systematically studying and further refining value-based pathways for patients presenting with injuries and other high ED spending conditions as well as those conditions with increased relative ED spending over time (e.g., biliary diseases) may be a high yield mechanism for assessing if such pathways affect ED spending trajectories. Conditions such as low back and neck pain, which showed relative growth and had the fifth highest level of ED spending in 2016, suggests another potential area for intervention, especially since there is poor compliance with evidence-based guidelines for musculoskeletal complaints [38–40].

This study’s disposition analysis suggests that important sources of ED spending are missed when studies focus only on the treat and release ED group, which are known to comprise the majority of ED visits in the U.S. [1, 41]. Exploring those conditions for which there is high ED spending among those who are ultimately admitted to the hospital, such as septicemia, can identify areas where intensive ED management can yield broader health system value (e.g., improved outcomes and lower overall hospitalization spending).

The primary focus of this descriptive study is to contextualize ED spending over time across a range of variables. However, we also took into account the well-known increase in ED visits over time [41] by showing ED spending per visit. We show modest growth in ED spending per visit over time and variation in this metric by health condition. This is consistent with prior studies suggesting that service price and intensity–as opposed to utilization–may play a more sizable role in ED spending trends [30, 42]. Spending due to increasing prices and intensity may allow the ED to better care for an increasingly complex patient population. For instance, Burke et al. [43] recently demonstrated improved mortality rates among the most severely ill patients treated in the ED, including the non-hospitalized population. However, the change in trajectory for ED spending on hypertension, which had initially been increasing but then had a downward trajectory in recent years, suggests that efforts to improve adherence to evidence-based practice may hold promise for mitigating some ED spending [44]. In the setting of this general tendency towards growth, more research is needed to elucidate if and where inefficiencies in ED care exist in order to inform strategic reductions in low-value spending.

This study provides a needed assessment of where ED spending has occurred over time, serving as a foundation for future work to explore the value of this spending, assessments of this growth, and whether it has been sufficient for supporting the unique role that the ED serves to millions of Americans. This is especially timely in the COVID-19 era where ED volumes have fluctuated dramatically [45, 46], thereby making ED spending a dynamic trend to monitor.

### Limitations

Though it utilizes a novel approach to provide a needed assessment of ED spending over time, this analysis and the study from which the estimates were drawn [18] are purely observational. This study is descriptive and aims to help guide future studies that may be able to assess underlying drivers of the observed spending trends (e.g., number of ED beds in a community, prices, intensity of services). While NEDS is the most comprehensive ED dataset available, its sampling strategy is limited to hospital-based EDs, thereby excluding freestanding EDs which may increase ED spending within healthcare markets [47]. Further, this analysis relies on a dataset that linked a randomly selected 50% sample of the available NEDS data to DEX health categories. Though efforts were made to account for this 50% sampling with survey weights in that prior analysis, and we demonstrate balance in observed characteristics and outcomes between the included versus excluded sample (see S1 Appendix), more data generally leads to more precise estimates. Because we were did not process the full NEDS dataset, we expect our estimates have larger uncertainty intervals. Additionally, NEDS is visit-based and not a patient-level analysis, thus we cannot isolate spending trends among high-utilizers, which have been the focus of many value-based initiatives [6]. Further, though established adjustments were made to accurately transform the facility-based charge variable to actual spending, these may be imperfect. For instance, NEDS data may not reliably attribute laboratory and radiology costs to certain ED codes, thereby biasing these ED estimates downward [48]. Also, the scope of this analysis was focused on ED spending, which fails to capture the downstream inpatient or ambulatory costs that are influenced heavily by ED providers [1]. Lastly, the disposition analysis showed that the majority of ED spending is attributable to what is known to be the majority population treated in the ED (those who are treated and released versus the relatively smaller proportion of patients that are admitted to the hospital through the ED). However, this analysis was limited in that it could not specifically account for volume due to how these data were processed. Future work that can isolate the specific ED costs generated per visit based on whether someone was discharged or admitted to the hospital through the ED is needed.

## Conclusions

ED spending is both sizable and growing in the U.S. in spite of policy interest to curb these trends. Though the ED accounts for only a fraction of national health spending, its growth relative to other sectors suggests the importance of delineating where these dollars have been allocated over time. Focusing on health conditions where the greatest absolute and relative changes in ED spending are occurring, even after accounting for increases in visits, can inform future value-based initiatives within the ED to ensure optimal care for the millions of patients it serves. Further, as ED volumes continue to fluctuate throughout the ongoing COVID-19 pandemic, it will be important for future work to assess this dynamic trend of ED spending.

## Supporting information

S1 Appendix Contains all supporting tables and figures. (DOCX)
