# Supplementary material for: Healthcare spending in U.S. emergency departments by health condition, 2006–2016
Source: PLoS One. 2021 Oct 27;16(10):e0258182. doi: 10.1371/journal.pone.0258182 (PMC8550368; doi:10.1371/journal.pone.0258182)
Supplement: S1 Appendix — (DOCX) [file pone.0258182.s001.docx]

**APPENDIX**

**Healthcare Spending in U.S. Emergency Departments by Health Condition, 2006-2016**

Short Title: U.S. Emergency Department Healthcare Spending, 2006-2016

Authors: Kirstin Woody Scott, MD, MPhil, PhD^1,2 #a^¶

Angela Liu, MPH^2,#b^¶

Carina Chen, MA^2^

Alexander S. Kaldjian, MS, MPH^2 #c^

Amber K. Sabbatini, MD, MPH^3^

Herbert C. Duber, MD, MPH^2,3^

Joseph L. Dieleman, PhD^2*^

Affiliations:

1. Department of Emergency Medicine, University of Michigan, Ann Arbor, MI, USA
2. Institute for Health Metrics and Evaluation, University of Washington, Seattle WA, USA
3. Department of Emergency Medicine, University of Washington, Seattle, WA, USA

Current address:

#a Prior affiliation: at the time of the project, Dr. Scott was a medical student at Harvard Medical School, Boston, MA, USA

#b Current address: Department of Health Policy and Management, Johns Hopkins Bloomberg School of Public Health, Baltimore, MD, USA

#c Current address: Bluesquare, Brussels, Belgium

¶ KWS and AL are Joint First Authors and contributed equally to the work

*Corresponding Author

Email: [dieleman@uw.edu](mailto:dieleman@uw.edu) (JD)

**Appendix Table of Contents**

[1. Conceptual Framework 3](#_Toc83764516)

[2. Analytic Dataset 3](#_Toc83764517)

[S1 Table. Comparison of Weighted Counts of ED Visits Between Published by NEDS versus DEX Processed Analytic Sample (All Years = 2006-2013, 2015-2016) 4](#_Toc83764518)

[S2 Table. Comparison of Analytic Sample and Excluded Observations, Unweighted and Weighted Analysis (All Years = 2006-2013, 2015-2016) 5](#_Toc83764519)

[3. Aggregated Health Categories 5](#_Toc83764520)

[S3 Table. Comparison of Aggregated Health Categories (Cause Groups) 7](#_Toc83764521)

[S4 Table. Mapping of 154 DEX Health Conditions to 15 Aggregated Health Categories 8](#_Toc83764522)

[4. Generating ED spending estimates in relation to national health care spending 12](#_Toc83764523)

[S1 Figure. Percent of National Health Care Spending Attributable to the Emergency Department, 2006 to 2016 13](#_Toc83764524)

[5. Generating relative changes in ED spending compared to other sectors 13](#_Toc83764525)

[S5 Table. Relative Growth in Health Care Spending Across All Sectors, 2006-2016 14](#_Toc83764526)

[6. Disaggregating ED Spending by Payer 14](#_Toc83764527)

[S2 Figure. Emergency Department Spending by Payer Over Time, 2006-2016 15](#_Toc83764528)

[S6 Table. Population-Standardized Annualized Rate of Change (AROC) from 2006-2016 by Payer for 15 Aggregated Health Categories 16](#_Toc83764529)

[7. Disaggregating ED Spending by Age-Sex Groups 16](#_Toc83764530)

[S3 Figure. Emergency Department Spending by Payer, Age, and Sex, 2006 and 2016 17](#_Toc83764531)

[8. Disposition Analysis 17](#_Toc83764532)

[S4 Figure. ED spending by age, disposition, and aggregate health category, 2016 18](#_Toc83764533)

[9. Volume Estimates 18](#_Toc83764534)

[S5 Figure. Estimates for Number of Emergency Department Encounters by Year 19](#_Toc83764535)

[10. Additional Results - ED Spending Per Visit Analysis 19](#_Toc83764536)

[S6 Figure. ED Spending Per Visit By the 15 Health Conditions Associated with the Highest Levels of ED Spending as of 2016 21](#_Toc83764537)

[11. Complete Tables 21](#_Toc83764538)

[S7 Table. Emergency Department (ED) Spending on Hospitalized Patients, 2016 21](#_Toc83764539)

[S8 Table. Total ED Spending, Absolute and Relative Changes in ED Spending, and Changes in ED Spending Per Visit, All Conditions 27](#_Toc83764540)

[12. Appendix References 37](#_Toc83764541)

# **Conceptual Framework**

This manuscript focused on emergency department (ED) spending serves as a companion piece to a publication by the U.S. Disease Expenditure (DEX) project at the Institute for Health Metrics and Evaluation that examines personal healthcare spending in the U.S. from 1996 through 2016.[1] Given contemporary policy debate surrounding the value of ED care and public concern regarding the cost of ED care, including the topic of surprise billing[2], a more in-depth analysis to evaluate trends in ED spending was merited.

This ED-focused manuscript relies mostly on the same dataset used for that broader, published analysis by Dieleman and colleagues[1], which is accompanied by a Supplementary Appendix. That extensive Supplementary Appendix provides greater detail regarding the microdata, including how the primary dataset used for this analysis (the Nationwide Emergency Department Sample (NEDS)) were formatted, processed, and adjusted to yield ED spending estimates.[1]

In addition, we summarize some of the important differences between this analysis and the prior published study. First, this analysis focuses on ED spending from 2006 through 2016 whereas the DEX dataset included spending estimates for various types of care, including the ED, back to 1996. This decision of limiting the current ED spending analysis from 2006 to 2016 was preferable since our primary data set, NEDS, also started in 2006; it was ideal to focus our results for trends in the past decade using a consistent data source.[3] Second, and related, this current study includes a unique ED disposition analysis (i.e. ED spending for those who were admitted to the hospital from the ED versus those who were treated and discharged from the ED), which is summarized further in this Appendix and required NEDS data, which started in 2006. Third, this study describes ED spending over time, whereas the DEX summary piece only showcases ED spending estimates from 2016. Fourth, though these ED spending summaries are by the same 154 conditions as described in the companion manuscript, the grouping of these health conditions into aggregate conditions varies slightly. The original DEX analysis grouped the 154 conditions into 14 aggregate health categories[1], whereas this paper uses 15 aggregate health conditions, which are more clinically relevant in the ED setting of care and are described further below. Fifth, this analysis is unique in that we provide not only spending trends but also account for spending by ED volume estimates from 2006 to 2016, allowing for an ED spending per visit analysis.

# **Analytic Dataset**

This ED spending manuscript primarily relies on the NEDS dataset, which is the largest available all-payer dataset for ED encounters in the United States and available for purchase from the Healthcare Cost Utilization Project (HCUP) (https://www.hcup-us.ahrq.gov/tech_assist/centdist.jsp). To compare how the analytic dataset would compare to the original raw files as available by NEDS, we summarize how data processing could have affected our initial volume estimates. To first process the dataset, any encounter that was missing age and sex information was dropped as this was required for DEX processing. This was a relatively small number of encounters per year in light of NEDS including a sample size of between 25 million – 32 million unweighted encounters over the years.[3] Due to the size of the NEDS dataset and the existing DEX infrastructure involved in reading and writing data files, a randomly selected half of the processed NEDS dataset was used to generate the spending estimates. We first showcase how our weighted national estimates for total ED visits nationally compare with the published weighted counts provided directly by HCUP (see **S1 Table**). This demonstrates that even when using the 50% sample, our weighted national counts of total ED visits were nearly equivalent to what HCUP publishes. In addition, in **S2 Table**, we compare the analytic sample to the excluded sample, which showed no substantive demographic (age and sex distribution) or outcome (raw ED charges and total hospitalization charges variables) differences across groups. Additionally, at the time of this analysis, the DEX project did not have access to 2014 NEDS data. As such, all estimates for 2014 values reflect the average of both 2013 and 2015 ED spending estimates by age, sex, cause, and disposition. One important qualification for providing estimates for 2014 is that the mean of the 2013 and 2015 estimates were used for the distribution of ED spending, but not the level of ED spending.

## S1 Table. Comparison of Weighted Counts of ED Visits Between Published by NEDS versus DEX Processed Analytic Sample (All Years = 2006-2013, 2015-2016)

|  | Completed NEDS Sample  (published on HCUP website)^a^ | Analytic Sample (50% of Formatted DEX Sample)^b^ | Difference in Weighted Counts  (%) |
| --- | --- | --- | --- |
| Weighted Counts for Total ED Visits Nationally | \| 1,313,795,188 \| \| --- \| \|  \| | \| 1,313,417,548 \| \| --- \| | 0.03% |

*Notes:* ^a^Sum of estimates shown in Table 1 of HCUP website - <https://www.hcup-us.ahrq.gov/db/nation/neds/NEDS_Introduction_2016.jsp> (excluding 2014). ^b^Formatted DEX sample = The raw NEDS data first had to be formatted, which includes a step that drops any encounters that were missing sex or age. The largest drop was in 2009 where >30k raw NEDS observations were missing sex (0.11% of the entire available sample).

## S2 Table. Comparison of Analytic Sample and Excluded Observations, Unweighted and Weighted Analysis (All Years = 2006-2013, 2015-2016)

|  | **Unweighted Analysis** | | **Weighted Analysis^a^** | |
| --- | --- | --- | --- | --- |
| *Sample* | Analytic Sample  (odd rows) | Excluded Sample  (even rows) | Analytic Sample  (odd rows) | Excluded Sample  (even rows) |
| *Number of Observations* | *n=145414874* | *n=145414667* | *n=1313417548* | *n=1313422496* |
|  | Mean (SD) or % | Mean (SD) or % | Mean (SD) or % | Mean (SD) or % |
| ED Charge | 2334.46 (3723.03) | 2334.14 (3715.57) | 2296.84 (3657.31) | 2296.56 (3650.84) |
| Total Charges for Admitted Patients | 37555.62 (61469.76) | 37555.96 (61574.42) | 37107.48 (61149.57) | 37109.96. (61244.73) |
| Age (mean) | 36.88 (24.11) | 36.87 (24.11) | 36.89 (24.14) | 36.89 (24.14) |
| Age Group (%) |  |  |  |  |
| <20 years | 23.78 | 23.78 | 23.82 | 23.82 |
| 21-44 years | 36.47 | 36.48 | 36.37 | 36.38 |
| 45-64 | 22.25 | 22.25 | 22.28 | 22.28 |
| 65+ | 17.5 | 17.49 | 17.53 | 17.53 |
| Sex (%) |  |  |  |  |
| Female | 55.29 | 55.29 | 55.16 | 55.17 |
| Male | 44.71 | 44.71 | 44.84 | 44.83 |
| Missing ED charge (%) | 16.39 | 16.39 | n/a | n/a |
| Admitted to Hospital (%) | 14.76 | 14.76 | 14.67 | 14.67 |

*Notes:* ^a^Weighted sample accounts for survey weights for each encounter and doubles the weight to produce national estimates since a 50% sample was used. These are raw ED charges that have not been processed (e.g., our study showcases ED spending in terms of payments and not charges) and do not yet account for inflation. The primary objective of this table is to showcase how the raw inputs compare in the included versus excluded 50% random sample.

# **Aggregated Health Categories**

The following section summarizes the 154 health conditions (which are mutually exclusive and collectively exhaustive) described by the Disease Expenditure (DEX) project and how they map to a smaller subset of aggregated health categories (or cause groups). Prior DEX work had mapped to a total of 14 aggregated health categories.[1] However, to ensure this list and the aggregation mapping are as clinically relevant to the ED setting of care as possible and future work, this list was slightly adjusted and then expanded to a total of 15 aggregated health categories (see comparison in **S3 Table**). Aside from terminology, none of the ICD code mapping was changed for the 154 DEX health conditions. We summarize the key changes between the mapping of the individual health conditions to the broader health categories as compared to prior published DEX work here. **S4 Table** lists the individual 154 health conditions and how each maps to one of the 15 aggregated health categories. For spending by each of these individual health conditions that makes up these broader categories (e.g. Injuries), please see **S8 Table**.

1. Individual health condition terminology changes (list of 154 health conditions):
   1. “Peripartum death due to complications of a preexisting medical condition” was changed to “indirect maternal complication of a preexisting medical condition”
   2. “Urinary diseases and male infertility” was changed to “urinary diseases”
   3. “Treatment of hyperlipidemia,” “treatment of hypertension,” and “treatment of obesity” was changed to “hyperlipidemia,” “hypertension,” and “morbid-obesity,” respectively
   4. “Exposure to mechanical forces” was changed to “unintentional injuries due to mechanical forces”
   5. “Other musculoskeletal disorders” was changed to “other musculoskeletal conditions, including joint pain”
   6. “Alzheimer’s disease and other dementias” was changed to “dementia”
   7. “Donor” was changed to “organ donation (harvesting)”
   8. “Well baby” was changed to “Well newborn care”
2. Changes in aggregated health categories (**S3 Table**). Prior published work for the DEX project featured 14 categories, however, this ED specific analysis utilized an updated mapping that now has the individual 154 health conditions aggregating to 15 health categories:

## S3 Table. Comparison of Aggregated Health Categories (Cause Groups)

| Current Study Version (n=15) | Prior Published Study[1] Listing (n=14) |
| --- | --- |
| 1. Cardiovascular diseases | 1. Cardiovascular diseases |
| 1. Chronic respiratory diseases | 2. Chronic respiratory diseases |
| 1. Other non-communicable diseases | 3. Other non-communicable diseases |
| 1. Digestive diseases | 4. Digestive diseases |
| 1. Injuries | 5. Injuries |
| 1. Behavioral health and substance use disorders | 6. Mental and behavioral disorders |
| 1. Musculoskeletal conditions | 7. Musculoskeletal disorders |
| 1. Cancers | 8. Neoplasms |
| 1. Neurological disorders | 9. Neurological disorders |
| 1. Communicable and nutrition disorders | 10. Communicable, maternal, neonatal, and nutrition disorders |
| 1. Maternal and neonatal conditions |  |
| 1. Diabetes and kidney diseases | 11. Diabetes, urogenital, blood, and endocrine diseases |
| 1. Endocrine disorders |  |
| 1. Skin and other sense organ disorders |  |
| 1. Prevention and coordination | 12. Expenditure on risk factors |
|  | 13. Well care |
|  | 14. Cirrhosis of the liver |

A detailed description of these changes noted in the above table is provided below:

- 1. “Diabetes, urogenital, blood, and endocrine diseases” was split into 3 aggregated categories: “Diabetes and kidney diseases,” “endocrine disorders,” and “other non-communicable diseases”
  2. “Communicable, maternal, neonatal, and nutritional disorders” was split into 2 aggregated categories: “Communicable and nutrition disorders” and “maternal and neonatal conditions”
  3. “Expenditure on risk factors” was changed to “prevention and coordination”
  4. “Mental and behavioral disorders” was changed to “Behavioral health and substance use disorders”
  5. “Neoplasms” was changed to “cancers”
  6. “Well care” was changed to “prevention and coordination”
  7. “Cirrhosis of the liver” was changed to “digestive diseases”
  8. “Skin and other sense organ disorders” was created and includes health conditions “sense organ diseases” and “skin and subcutaneous diseases” which were originally aggregated into “other non-communicable diseases”

## S4 Table. Mapping of 154 DEX Health Conditions to 15 Aggregated Health Categories

| **#** | **Individual Health Conditions (n=154)** | **Aggregated Health Category (n=15)** |
| --- | --- | --- |
| 1 | Atrial fibrillation and flutter | Cardiovascular diseases |
| 2 | Aortic aneurysm | Cardiovascular diseases |
| 3 | Cardiomyopathy and myocarditis | Cardiovascular diseases |
| 4 | Endocarditis | Cardiovascular diseases |
| 5 | Heart Failure | Cardiovascular diseases |
| 6 | Hypertensive heart disease | Cardiovascular diseases |
| 7 | Ischemic heart disease | Cardiovascular diseases |
| 8 | Other cardiovascular and circulatory diseases | Cardiovascular diseases |
| 9 | Peripheral vascular disease | Cardiovascular diseases |
| 10 | Rheumatic heart disease | Cardiovascular diseases |
| 11 | Cerebrovascular disease | Cardiovascular diseases |
| 12 | Asthma | Chronic respiratory diseases |
| 13 | Chronic obstructive pulmonary disease | Chronic respiratory diseases |
| 14 | Interstitial lung disease and pulmonary sarcoidosis | Chronic respiratory diseases |
| 15 | Other chronic respiratory diseases | Chronic respiratory diseases |
| 16 | Pneumoconiosis | Chronic respiratory diseases |
| 17 | Cirrhosis of the liver | Digestive diseases |
| 18 | Neglected tropical diseases and malaria | Communicable and nutrition disorders |
| 19 | Diarrheal diseases | Communicable and nutrition disorders |
| 20 | Diphtheria | Communicable and nutrition disorders |
| 21 | Encephalitis | Communicable and nutrition disorders |
| 22 | Hepatitis | Communicable and nutrition disorders |
| 23 | HIV/AIDS | Communicable and nutrition disorders |
| 24 | Other infectious diseases | Communicable and nutrition disorders |
| 25 | Intestinal infectious diseases | Communicable and nutrition disorders |
| 26 | Leprosy | Communicable and nutrition disorders |
| 27 | Lower respiratory tract infections | Communicable and nutrition disorders |
| 28 | Complications of abortion | Maternal and neonatal conditions |
| 29 | Maternal hemorrhage | Maternal and neonatal conditions |
| 30 | Hypertensive conditions of pregnancy | Maternal and neonatal conditions |
| 31 | Indirect maternal complication of a preexisting medical condition | Maternal and neonatal conditions |
| 32 | Obstructed labor | Maternal and neonatal conditions |
| 33 | Other maternal disorders | Maternal and neonatal conditions |
| 34 | Maternal sepsis and other pregnancy related infection | Maternal and neonatal conditions |
| 35 | Measles | Communicable and nutrition disorders |
| 36 | Meningitis | Communicable and nutrition disorders |
| 37 | Neonatal encephalopathy (birth asphyxia and birth trauma) | Maternal and neonatal conditions |
| 38 | Hemolytic disease in fetus and newborn and other neonatal jaundice | Maternal and neonatal conditions |
| 39 | Other neonatal disorders | Maternal and neonatal conditions |
| 40 | Preterm birth complications | Maternal and neonatal conditions |
| 41 | Sepsis and other infectious conditions of the newborn baby | Maternal and neonatal conditions |
| 42 | Iodine deficiency | Communicable and nutrition disorders |
| 43 | Iron-deficiency anemia | Communicable and nutrition disorders |
| 44 | Other nutritional deficiencies | Communicable and nutrition disorders |
| 45 | Protein-energy malnutrition | Communicable and nutrition disorders |
| 46 | Vitamin A deficiency | Communicable and nutrition disorders |
| 47 | Otitis media | Communicable and nutrition disorders |
| 48 | Septicemia | Communicable and nutrition disorders |
| 49 | Sexually transmitted diseases excluding HIV | Communicable and nutrition disorders |
| 50 | Tuberculosis | Communicable and nutrition disorders |
| 51 | Tetanus | Communicable and nutrition disorders |
| 52 | Upper respiratory tract infections | Communicable and nutrition disorders |
| 53 | Varicella | Communicable and nutrition disorders |
| 54 | Whooping cough | Communicable and nutrition disorders |
| 55 | Chronic kidney diseases | Diabetes and kidney diseases |
| 56 | Diabetes mellitus | Diabetes and kidney diseases |
| 57 | Endocrine, metabolic, blood, and immune conditions | Endocrine disorders |
| 58 | Acute glomerulonephritis | Diabetes and kidney diseases |
| 59 | Gynecological diseases | Other non-communicable diseases |
| 60 | Hemoglobinopathies and hemolytic anemias | Other non-communicable diseases |
| 61 | Acute renal failure | Diabetes and kidney diseases |
| 62 | Urinary diseases | Other non-communicable diseases |
| 63 | Appendicitis | Digestive diseases |
| 64 | Gallbladder and biliary diseases | Digestive diseases |
| 65 | Gastritis and duodenitis | Digestive diseases |
| 66 | Inguinal or femoral hernia | Digestive diseases |
| 67 | Inflammatory bowel disease | Digestive diseases |
| 68 | Paralytic ileus and intestinal obstruction | Digestive diseases |
| 69 | Other digestive diseases | Digestive diseases |
| 70 | Pancreatitis | Digestive diseases |
| 71 | Peptic ulcer disease | Digestive diseases |
| 72 | Vascular intestinal conditions | Digestive diseases |
| 73 | Hyperlipidemia | Cardiovascular diseases |
| 74 | Hypertension | Cardiovascular diseases |
| 75 | Morbid-obesity | Endocrine disorders |
| 76 | Tobacco cessation | Prevention and coordination |
| 77 | Animal contact | Injuries |
| 78 | Exposure to forces of nature | Injuries |
| 79 | Drowning | Injuries |
| 80 | Falls | Injuries |
| 81 | Fire, heat and hot substances | Injuries |
| 82 | Foreign body | Injuries |
| 83 | Interpersonal violence | Injuries |
| 84 | Unintentional injuries due to mechanical forces | Injuries |
| 85 | Other unintentional injuries | Injuries |
| 86 | Poisonings | Injuries |
| 87 | Self-harm | Injuries |
| 88 | Other transport injuries | Injuries |
| 89 | Road injuries | Injuries |
| 90 | Collective violence and legal intervention | Injuries |
| 91 | Attention-deficit/hyperactivity disorder | Behavioral health and substance use disorders |
| 92 | Alcohol use disorders | Behavioral health and substance use disorders |
| 93 | Anxiety disorders | Behavioral health and substance use disorders |
| 94 | Bipolar disorder | Behavioral health and substance use disorders |
| 95 | Conduct disorder | Behavioral health and substance use disorders |
| 96 | Other substance use disorders | Behavioral health and substance use disorders |
| 97 | Eating disorders | Behavioral health and substance use disorders |
| 98 | Idiopathic intellectual disability | Behavioral health and substance use disorders |
| 99 | Other mental and behavioral disorders | Behavioral health and substance use disorders |
| 100 | Autistic spectrum disorders | Behavioral health and substance use disorders |
| 101 | Schizophrenia | Behavioral health and substance use disorders |
| 102 | Depressive disorders | Behavioral health and substance use disorders |
| 103 | Gout | Musculoskeletal conditions |
| 104 | Osteoarthritis | Musculoskeletal conditions |
| 105 | Other musculoskeletal conditions, including joint pain | Musculoskeletal conditions |
| 106 | Low back and neck pain | Musculoskeletal conditions |
| 107 | Rheumatoid arthritis | Musculoskeletal conditions |
| 108 | Bladder cancer | Cancers |
| 109 | Brain and nervous system cancers | Cancers |
| 110 | Breast cancer | Cancers |
| 111 | Cervical cancer | Cancers |
| 112 | Colon and rectum cancers | Cancers |
| 113 | Esophageal cancer | Cancers |
| 114 | Gallbladder and biliary tract cancer | Cancers |
| 115 | Hodgkin lymphoma | Cancers |
| 116 | Kidney cancer | Cancers |
| 117 | Larynx cancer | Cancers |
| 118 | Leukemia | Cancers |
| 119 | Liver cancer | Cancers |
| 120 | Trachea, bronchus, and lung cancers | Cancers |
| 121 | Non-Hodgkin lymphoma | Cancers |
| 122 | Malignant skin melanoma | Cancers |
| 123 | Mouth cancer | Cancers |
| 124 | Multiple myeloma | Cancers |
| 125 | Nasopharynx cancer | Cancers |
| 126 | Non-melanoma skin cancer | Cancers |
| 127 | Other cancers | Cancers |
| 128 | Other pharynx cancer | Cancers |
| 129 | Ovarian cancer | Cancers |
| 130 | Pancreatic cancer | Cancers |
| 131 | Prostate cancer | Cancers |
| 132 | Stomach cancer | Cancers |
| 133 | Testicular cancer | Cancers |
| 134 | Thyroid cancer | Cancers |
| 135 | Uterine cancer | Cancers |
| 136 | dementia | Neurological disorders |
| 137 | Epilepsy | Neurological disorders |
| 138 | Migraine | Neurological disorders |
| 139 | Multiple sclerosis | Neurological disorders |
| 140 | Other neurological conditions | Neurological disorders |
| 141 | Parkinson's disease | Neurological disorders |
| 142 | Tension-type headache | Neurological disorders |
| 143 | Oral disorders | Other non-communicable diseases |
| 144 | Sense organ diseases | Skin and other sense organ disorders |
| 145 | Congenital anomalies | Other non-communicable diseases |
| 146 | Skin and subcutaneous diseases | Skin and other sense organ disorders |
| 147 | Counselling services | Prevention and coordination |
| 148 | Organ donation (harvesting) | Prevention and coordination |
| 149 | Family planning | Maternal and neonatal conditions |
| 150 | Social services | Prevention and coordination |
| 151 | Well dental | Prevention and coordination |
| 152 | Well newborn care | Maternal and neonatal conditions |
| 153 | Well person | Prevention and coordination |
| 154 | Pregnancy and postpartum care | Maternal and neonatal conditions |

# **Generating ED spending estimates in relation to national health care spending**

In order to determine both total ED spending by year and also the percentage of overall healthcare spending it accounts for, published National Health Expenditure Accounts (NHEA) data were realigned to generate total ED spending.[4] The National Health Expenditure Accounts (NHEA) data capture annual health spending by type of care, among other aggregate groupings. Specifically, NHEA provides estimates of spending in the following categories “Physician and Clinical Services” and “Hospital Care”, and these spending categories were redistributed into three settings of care, including ED (the primary outcome of interest in this analysis), inpatient, and outpatient spending. This redistribution relies on the Medical Expenditure Panel Survey (MEPS) data[5], which include panels across all three applicable settings of care (ED, inpatient, and outpatient), to parse out spending for each of the 3 settings from the two original NHEA categories. Since MEPS provides one overall charge (regardless of whether an admitted patient originated from the ED setting of care or elsewhere), an adjustment was made to account for patients who were treated in the ED and then hospitalized in the inpatient setting. Of note, NEDS provides an ED-specific charge for each encounter, regardless of disposition; only those encounters who are admitted to the hospital generate a separate overall charge that is inclusive of inpatient and ED spending. For this adjustment, we relied on taking the fraction of the original NEDS ED charge as noted above and an additional NEDS variable (TOTCHG_IP; which provides an overall sum of charges for patients hospitalized through the ED, inclusive of both their inpatient and ED charges), and reallocating spending to the ED that would have originally been entirely attributed to the inpatient setting. **S1 Figure** displays how ED spending as a percentage of national health care spending has changed from 2006 to 2016.

## S1 Figure. Percent of National Health Care Spending Attributable to the Emergency Department, 2006 to 2016


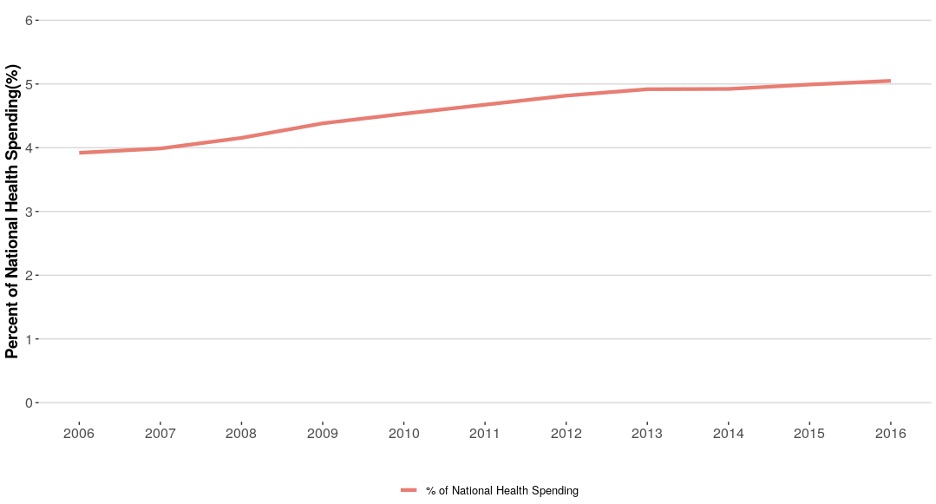


# **Generating relative changes in ED spending compared to other sectors**

The DEX project estimates personal health care spending across 7 services (ambulatory, dental care, emergency department, administration, inpatient care, long-term care, and pharmaceuticals). The published update in 2020 featured changes from 1996 to 2016.[1] However, using these data from the companion study, this ED-specific piece generated population adjusted annualized rates of change (AROC) for the entire health care economy (1.4% [95% CI, 1.4%-1.4%]) and all sectors, including the ED (4.4% [95% CI, 4.4-4.4%]). The population-adjusted AROCs for each service between 2006 and 2016 are shown in **S5 Table**.

## S5 Table. Relative Growth in Health Care Spending Across All Sectors, 2006-2016

| **Function** | **Population-Adjusted Annualized Rate of Change**  **(2006-2016)** | **95% Confidence Interval** |
| --- | --- | --- |
| All Sectors | **1.4%** | **(1.4%-1.4%)** |
| *Ambulatory* | 2.3% | (2.2%-2.3%) |
| *Dental Care* | 0.4% | (0.4%-0.5%) |
| *Emergency Department* | 4.4% | (4.4%-4.4%) |
| *General Administration* | 1.7% | (1.7%-1.7%) |
| *Inpatient* | 0.8% | (0.7%-0.8%) |
| *Long Term Care* | 0.2% | (0.2%-0.2%) |
| *Pharmaceuticals* | 0.7% | (0.7%-0.8%) |

# **Disaggregating ED Spending by Payer**

ED spending estimates were delineated by 3 payer groups: (1) private, (2) public, an (3) out-of-pocket (OOP). The “payer split” analysis completed in this study (and the companion broader health care spending study published by DEX)[1] relies on the Medical Expenditure Panel Survey (MEPS) dataset. MEPS provides information on payer relationships across a number of different types of care, including ED care.[6] Using centered-log transformations to ensure that the three payer ratios added up to exactly 1, a regression was performed for each single cause and payer, with age and year splines. This process is further described in the extensive Supplementary Appendix that accompanied the published DEX spending paper.[1]

The disaggregated spending estimates are scaled to total spending by payer as determined by the NHEA envelope (see Appendix Section 4). Because the NHEA envelope does not publish spending by the 3 payers described in this paper, the published NHEA categories must be realigned. To do this, public spending is determined by aggregating spending for Medicare, Medicaid, and other federal, state, and local programs, including Indian health services and Veteran Affairs. Private spending includes spending by private insurance, workers compensation, and worksite health care, among other categories, including “other private revenues” and “general assistance.” Finally, OOP spending is the spending from published NHEA category “oop”.

**S2 Figure** shows ED spending by payer from 2006 to 2016. **S6 Table** provides a synthesis of the annualized rate of growth for all conditions, as well as the 15 aggregated health categories, by payer.

## S2 Figure. Emergency Department Spending by Payer Over Time, 2006-2016


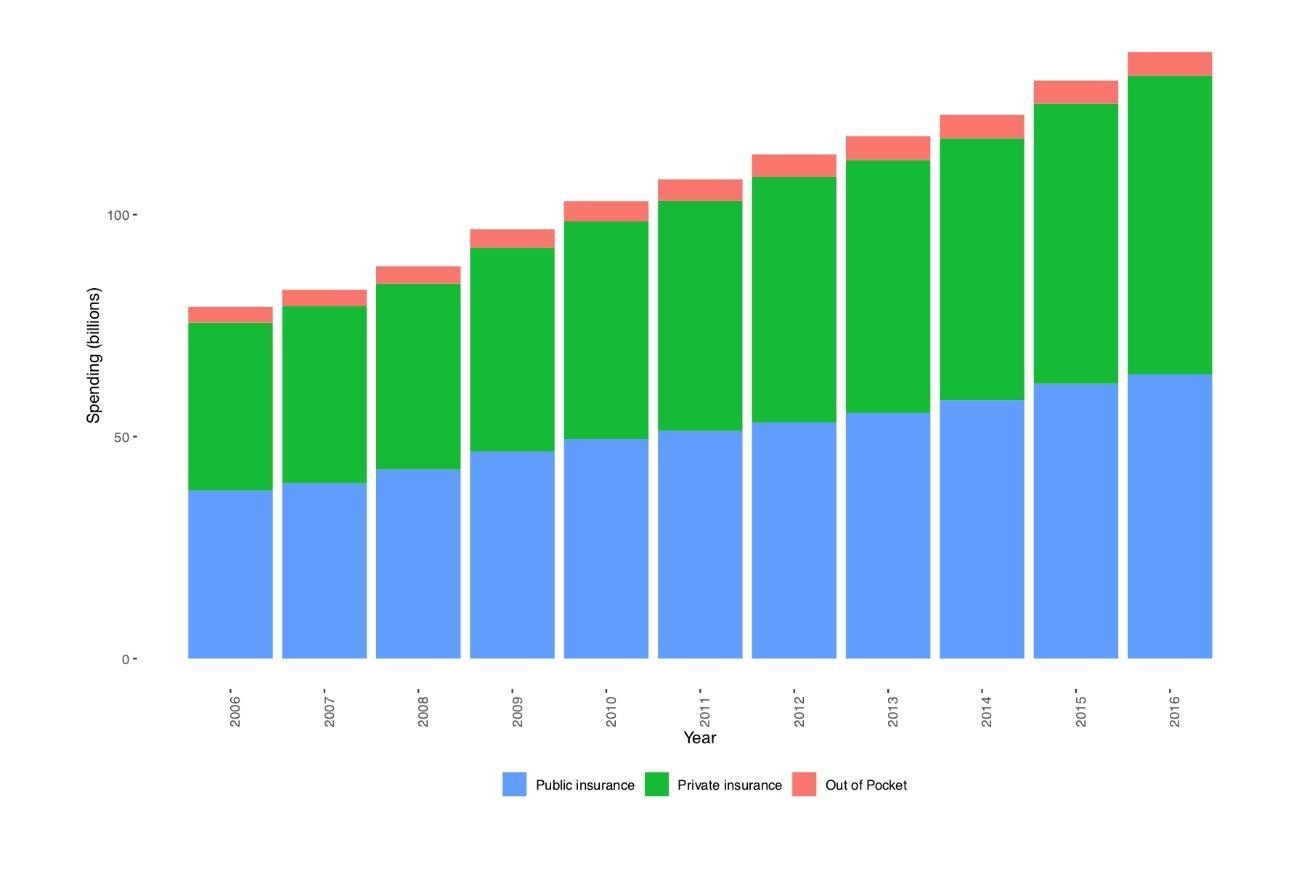


## S6 Table. Population-Standardized Annualized Rate of Change (AROC) from 2006-2016 by Payer for 15 Aggregated Health Categories

|  |  | Population-Standardized Annualized Rate of Change 2006-2016 | | | |
| --- | --- | --- | --- | --- | --- |
|  |  | Total | Public Insurance | Private Insurance | Out-of-pocket |
|  | All Conditions | 4.4 | 3.8 | 5.2 | 3.4 |
|  | Aggregated health categories |  |  |  |  |
| 1 | Endocrine disorders | 8.5 | 8.1 | 8.5 | 7.8 |
| 2 | Digestive diseases | 5.8 | 3.7 | 7.7 | 5.6 |
| 3 | Musculoskeletal conditions | 5.6 | 5.9 | 5.3 | 6.0 |
| 4 | Chronic respiratory diseases | 5.6 | 5.1 | 6.4 | 3.5 |
| 5 | Maternal and neonatal conditions | 5.6 | 3.4 | 9.4 | 8.4 |
| 6 | Neurological disorders | 5.3 | 3.0 | 8.1 | 5.7 |
| 7 | Behavioral health and substance use disorders | 5.3 | 5.1 | 6.0 | 2.5 |
| 8 | Skin and other sense organ disorders | 5.2 | 5.3 | 5.5 | -0.3 |
| 9 | Cancers | 4.9 | 2.6 | 9.0 | 8.9 |
| 10 | Other non-communicable diseases | 4.9 | 4.5 | 5.1 | 4.9 |
| 11 | Diabetes and kidney diseases | 4.8 | 2.9 | 10.9 | 7.6 |
| 12 | Prevention and coordination | 4.0 | 4.9 | 3.2 | -2.2 |
| 13 | Communicable and nutrition disorders | 3.7 | 4.9 | 2.4 | 1.0 |
| 14 | Injuries | 3.6 | 4.2 | 3.3 | 1.8 |
| 15 | Cardiovascular diseases | 2.1 | 0.4 | 5.6 | 1.7 |

*Note:* Population-standardized annualized rates of change (AROC) of Emergency Department (ED) spending across all payers, and then stratified by each payer group (public, private, and out-of-pocket (OOP)) for the 15 aggregated health causes (health categories).

# **Disaggregating ED Spending by Age-Sex Groups**

**S3 Figure** illustrates ED spending by the 38 age-sex groups analyzed in this study. Given that trends were similar when looking at less granular age group bins, the main text provides an overview by larger age bins than what is shown here. Further, this displays how ED spending by payer varies by age-sex groups.

## S3 Figure. Emergency Department Spending by Payer, Age, and Sex, 2006 and 2016


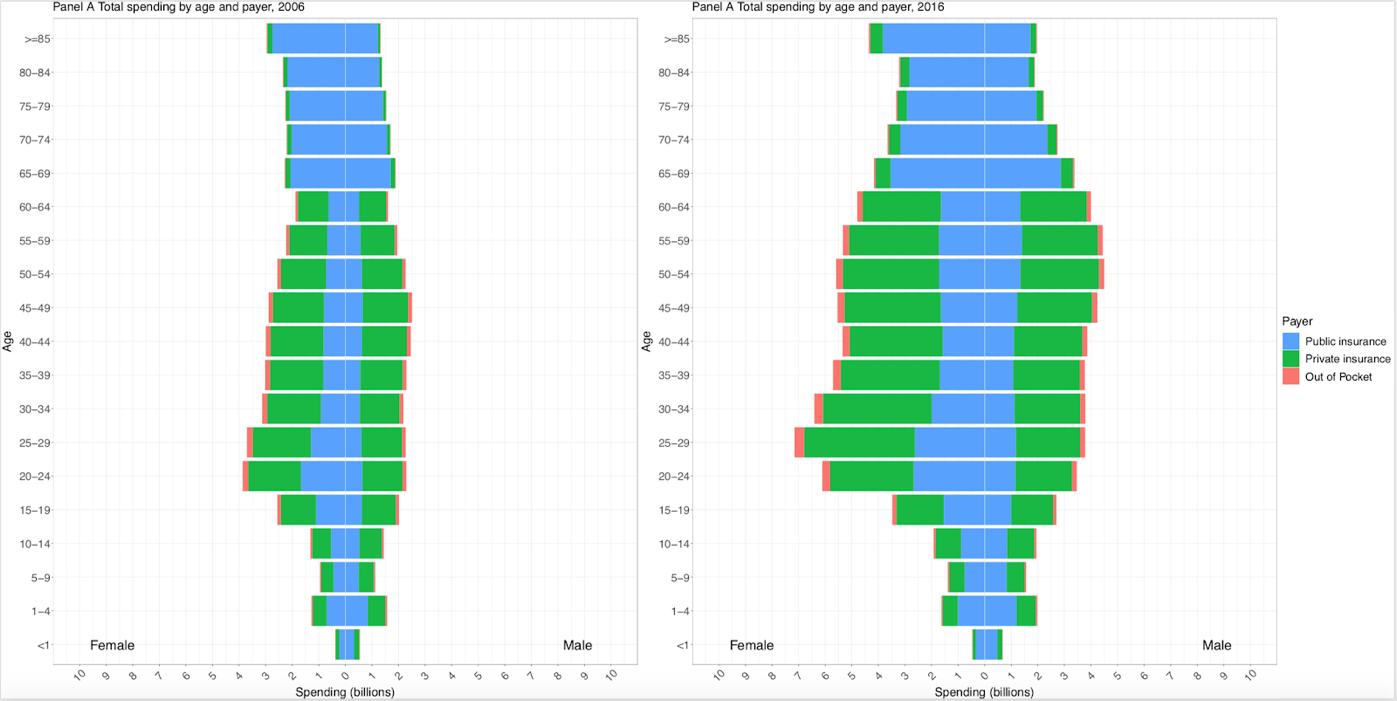


# **Disposition Analysis**

The ED spending estimates for this analysis are inclusive of all individuals who sought ED care, regardless of their ultimate disposition from the ED. Prior DEX estimates had been limited to including only ED visits that were in the “treat and release” category of patients and thus was an underestimate of ED spending for those patients who were treated in the ED and were admitted through the ED to the hospital.[7] The ED spending for those encounters who were treated in the ED and then were admitted to the hospital would have simply been attributed to the inpatient spending in prior work.[7] Other studies have aimed to contextualize ED spending as “episodes of care” and include both ED and inpatient spending, which are helpful but add further complexity to generating estimates for spending that should be attributed only to the ED.[8] Thus a strength of the updated DEX analysis was that it aimed to account for this known bias of inflating inpatient spending with spending that was known to have occurred in the ED but difficult to parse out with prior datasets.[1]

The current analysis complements the prior published study by delineating ED spending by these two key groups of encounters: 1) those patients that were discharged from the ED (i.e., “treat-and-release” or “discharged” encounters) versus 2) those patients admitted to a hospital following their ED evaluation (i.e., “admitted” encounters). Thus, the ED estimates presented in this paper capture comprehensive ED spending (i.e. among all ED visits). While these refined ED estimates are policy relevant and important, it is also helpful to understand spending in the ED for patients who were discharged versus admitted. Please note that this analysis was not able to isolate “observation” stays due to data limitations. NEDS reports that if a patient was treated in the ED, transferred to observation, and then was discharged home, then this encounter would fall into the treat and release file. Alternatively, if the patient was treated in the ED, transferred to observation, and then admitted, then this encounter would fall into the “inpatient” file. Regardless, each encounter would have an “ED charge” provided by NEDS, which is an estimate of the charges generated by the ED regardless of the patient’s disposition.

To split comprehensive ED spending, the NEDS dataset was used to calculate the proportion of ED spending for those who were discharged. Using the entire population in the NEDS data set, the numerator with ED spending for those who were discharged, and the denominator was ED spending for all patients (those who were either discharged or admitted). To apply these proportions, the proportions by age, sex, year, payer, and draw were multiplied with the comprehensive ED estimates to capture ED spending who were discharged. The difference between comprehensive ED estimates and discharged estimates was then used to calculate the ED spending estimates for those ultimately admitted. These proportions were then used to split the comprehensive ED estimates by health condition, age, sex, year, payer, and draw. **S4** **Figure** illustrates how ED spending varies by age group, disposition, and the 15-aggregated health categories (which are inclusive of all 154 individual health conditions considered in this analysis).

## S4 Figure. ED spending by age, disposition, and aggregate health category, 2016


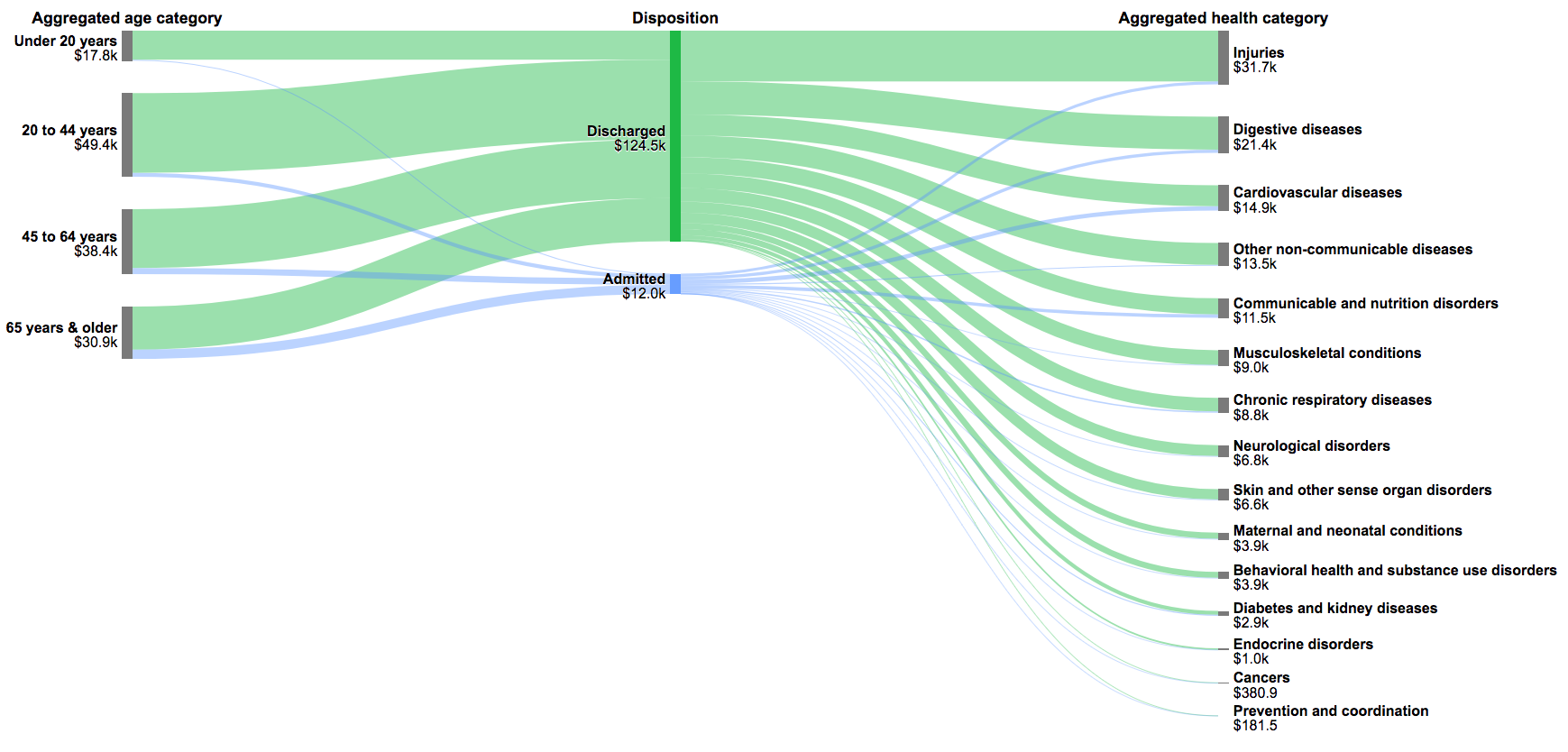


*Note:* For the middle section of ED Disposition, “Discharged” refers to ED encounters who were not admitted to the hospital (treat and release subgroup) whereas “Admitted” refers to encounters who started in the ED and were ultimately hospitalized.

# **Volume Estimates**

A unique contribution of this analysis relative to the prior DEX study is that estimates for ED volume are generated from 2006 to 2016 and by the 154 DEX health conditions, patient age, and patient sex using only the NEDS dataset. The process for generating final volume estimates is completed during the “smoothing” process that is detailed in the prior DEX study’s Supplement.[1] **S5 Figure** illustrates the estimated number of ED encounters over time. This demonstrates well-known and documented trends in the NEDS dataset of the growth of ED encounters over time and approximates those published national estimates of ED encounters as provided by the Healthcare Cost and Utilization Project (HCUP) NEDS dataset.[3]

## S5 Figure. Estimates for Number of Emergency Department Encounters by Year

1. Dieleman JL, Cao J, Chapin A, Chen C, Li Z, Liu A, et al. US Health Care Spending by Payer and Health Condition, 1996-2016. JAMA. 2020;323: 863–884. doi:10.1001/jama.2020.0734

2. Kliff S. Surprise Medical Bills, the High Cost of Emergency Department Care, and the Effects on Patients. JAMA Intern Med. 2019;179: 1457–1458. doi:10.1001/jamainternmed.2019.3448

3. Healthcare Cost and Utilization Project (HCUP). Nationwide Emergency Department Sample (NEDS), 2016. 2016 [cited 18 Nov 2019]. Available: https://www.hcup-us.ahrq.gov/db/nation/neds/NEDS_Introduction_2016.jsp

4. Centers for Medicare & Medicaid Services. National Health Expenditure Data. [cited 1 Feb 2020]. Available: https://www.cms.gov/Research-Statistics-Data-and-Systems/Statistics-Trends-and-Reports/NationalHealthExpendData

5. Medical Expenditure Panel Survey. Household Component Summary Data Tables. In: Emergency Room Services-Median and Mean Expenses per Person With Expense and Distribution of Expenses by Source of Payment: United States, 2014 [Internet]. [cited 1 Feb 2020]. Available: https://meps.ahrq.gov/mepsweb/data_stats/download_data_files.jsp

6. Agency for Healthcare Research and Quality. Medical Expenditure Panel Survey. [cited 1 Feb 2020]. Available: https://www.meps.ahrq.gov/mepsweb/

7. Dieleman JL, Baral R, Birger M, Bui AL, Bulchis A, Chapin A, et al. US Spending on Personal Health Care and Public Health, 1996-2013. JAMA. 2016;316: 2627–2646. doi:10.1001/jama.2016.16885

8. Galarraga JE, Pines JM. Costs of ED episodes of care in the United States. The American Journal of Emergency Medicine. 2016;34: 357–365. doi:10.1016/j.ajem.2015.06.001

# **Additional Results - ED Spending Per Visit Analysis**

Though the primary focus of this study was to describe ED spending over time and where it has been allocated across age, sex, payer, and health condition, it was merited to explore an important potential driver for ED spending: ED visits. The volume analysis is described earlier in this Appendix (see Section 3).

**S6 Figure** summarizes how ED spending per visit varies by the 15 health conditions associated with the highest level of ED spending as of 2016. For ease of interpretation, the main text focuses on the 5 conditions associated with the highest levels of ED spending as of 2016 (which account for 28% of all ED spending that year). However, a broader range of conditions helps to showcase the exception noted in the main manuscript – treatment of hypertension – and how it did not monotonically increase over time.

## S6 Figure. ED Spending Per Visit By the 15 Health Conditions Associated with the Highest Levels of ED Spending as of 2016


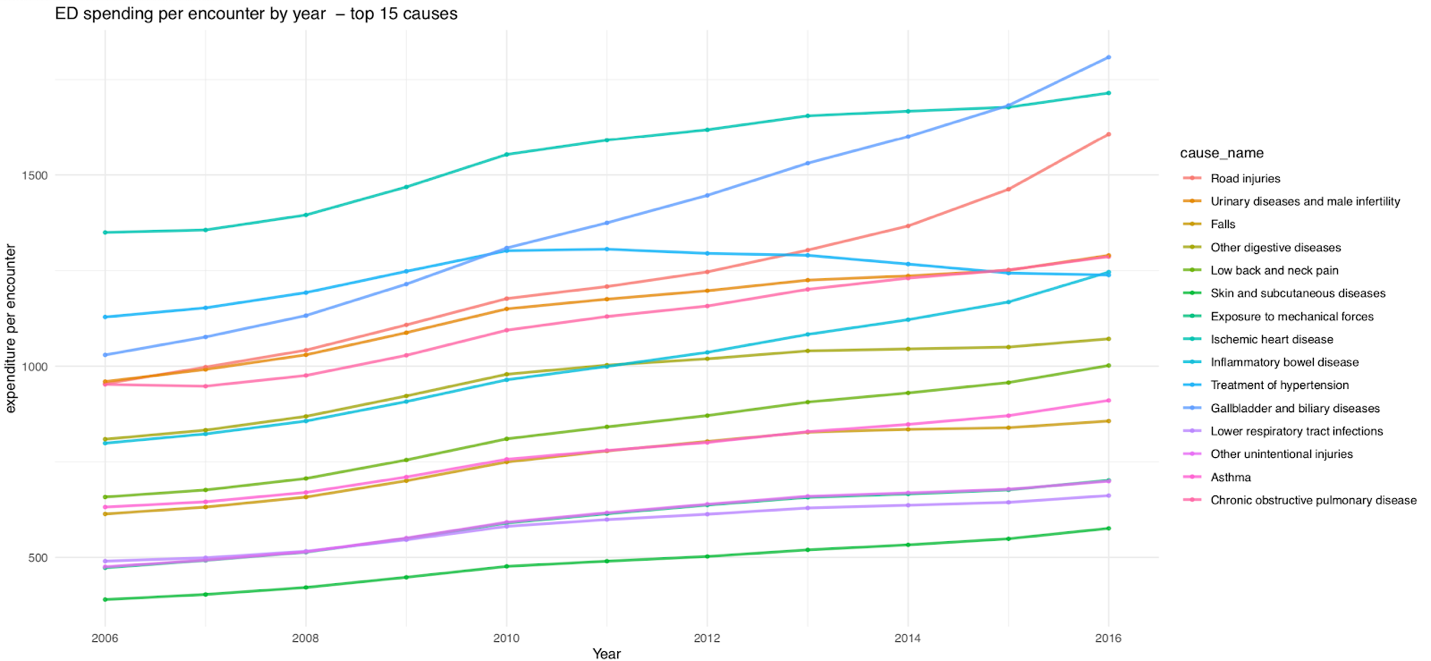


# **Complete Tables**

The following Tables showcase key results by the 154 individual DEX health conditions.

## S7 Table. Emergency Department (ED) Spending on Hospitalized Patients, 2016

| # | Condition | Admitted Group  Millions ($) | Treat and Release Group  Millions ($) | Total ED Spending  Millions ($) | ED Spending for Admitted Group  (%) |
| --- | --- | --- | --- | --- | --- |
| 1 | Septicemia | 957.7 | 172.4 | 1,130.0 | 84.7 |
| 2 | Ischemic heart disease | 821.8 | 3,767.1 | 4,589.0 | 17.9 |
| 3 | Falls | 622.0 | 7,837.3 | 8,459.3 | 7.4 |
| 4 | Lower respiratory tract infections | 566.4 | 3,558.3 | 4,124.7 | 13.7 |
| 5 | Heart Failure | 556.3 | 982.8 | 1,539.1 | 36.1 |
| 6 | Chronic obstructive pulmonary disease | 553.3 | 2,755.3 | 3,308.5 | 16.7 |
| 7 | Urinary diseases | 484.7 | 8,924.2 | 9,408.9 | 5.2 |
| 8 | Road injuries | 475.4 | 9,404.5 | 9,879.9 | 4.8 |
| 9 | Cerebrovascular disease | 469.7 | 1,254.5 | 1,724.3 | 27.2 |
| 10 | Gallbladder and biliary diseases | 405.9 | 3,749.8 | 4,155.7 | 9.8 |
| 11 | Skin and subcutaneous diseases | 405.2 | 4,607.4 | 5,012.6 | 8.1 |
| 12 | Cirrhosis of the liver | 376.7 | 1,006.3 | 1,383.0 | 27.2 |
| 13 | Diabetes mellitus | 357.0 | 1,980.9 | 2,338.0 | 15.3 |
| 14 | Atrial fibrillation and flutter | 295.6 | 1,156.2 | 1,451.8 | 20.4 |
| 15 | Other digestive diseases | 290.7 | 5,175.8 | 5,466.5 | 5.3 |
| 16 | Inflammatory bowel disease | 281.4 | 4,290.1 | 4,571.5 | 6.2 |
| 17 | Acute renal failure | 203.5 | 122.0 | 325.6 | 62.5 |
| 18 | Treatment of hypertension | 200.0 | 4,240.2 | 4,440.3 | 4.5 |
| 19 | Paralytic ileus and intestinal obstruction | 181.6 | 478.7 | 660.4 | 27.5 |
| 20 | Other musculoskeletal disorders | 169.2 | 3,121.0 | 3,290.2 | 5.1 |
| 21 | Diarrheal diseases | 150.9 | 597.2 | 748.1 | 20.2 |
| 22 | Asthma | 149.7 | 3,368.9 | 3,518.6 | 4.3 |
| 23 | Other cardiovascular and circulatory diseases | 147.9 | 755.9 | 903.8 | 16.4 |
| 24 | Other unintentional injuries | 143.5 | 3,411.0 | 3,554.5 | 4.0 |
| 25 | Depressive disorders | 133.0 | 803.6 | 936.7 | 14.2 |
| 26 | Iron-deficiency anemia | 128.1 | 522.9 | 651.0 | 19.7 |
| 27 | Low back and neck pain | 123.3 | 5,051.9 | 5,175.2 | 2.4 |
| 28 | Poisonings | 122.5 | 654.2 | 776.7 | 15.8 |
| 29 | Appendicitis | 122.4 | 2,943.4 | 3,065.8 | 4.0 |
| 30 | Endocrine, metabolic, blood, and immune disorders | 119.8 | 918.1 | 1,038.0 | 11.5 |
| 31 | Pregnancy and postpartum care | 115.3 | 284.7 | 399.9 | 28.8 |
| 32 | Exposure to mechanical forces | 114.6 | 4,643.7 | 4,758.3 | 2.4 |
| 33 | Other neurological disorders | 106.9 | 2,865.8 | 2,972.7 | 3.6 |
| 34 | Alzheimer's disease and other dementias | 103.9 | 865.9 | 969.7 | 10.7 |
| 35 | Pancreatitis | 91.1 | 305.1 | 396.2 | 23.0 |
| 36 | Peripartum death due to complications of a preexisting medical condition | 82.4 | 1,490.5 | 1,573.0 | 5.2 |
| 37 | Interpersonal violence | 75.6 | 2,017.8 | 2,093.5 | 3.6 |
| 38 | Other infectious diseases | 73.9 | 1,120.9 | 1,194.8 | 6.2 |
| 39 | Other chronic respiratory diseases | 65.4 | 1,858.8 | 1,924.1 | 3.4 |
| 40 | Anxiety disorders | 65.2 | 1,543.5 | 1,608.7 | 4.1 |
| 41 | Epilepsy | 63.1 | 808.7 | 871.8 | 7.2 |
| 42 | Chronic kidney diseases | 61.3 | 179.2 | 240.6 | 25.5 |
| 43 | Gastritis and duodenitis | 59.1 | 948.9 | 1,008.1 | 5.9 |
| 44 | Bipolar disorder | 57.6 | 207.5 | 265.1 | 21.7 |
| 45 | Gynecological diseases | 53.0 | 3,105.3 | 3,158.3 | 1.7 |
| 46 | Sense organ diseases | 52.3 | 1,520.5 | 1,572.7 | 3.3 |
| 47 | Drug use disorders | 49.4 | 483.7 | 533.1 | 9.3 |
| 48 | Migraine | 47.7 | 1,900.3 | 1,947.9 | 2.4 |
| 49 | Trachea, bronchus, and lung cancers | 47.3 | 69.0 | 116.3 | 40.7 |
| 50 | Alcohol use disorders | 46.2 | 412.0 | 458.2 | 10.1 |
| 51 | Peptic ulcer disease | 42.6 | 122.6 | 165.3 | 25.8 |
| 52 | Upper respiratory tract infections | 42.2 | 2,691.0 | 2,733.2 | 1.5 |
| 53 | Inguinal or femoral hernia | 31.1 | 469.3 | 500.3 | 6.2 |
| 54 | Other maternal disorders | 29.7 | 531.1 | 560.8 | 5.3 |
| 55 | Complications of abortion | 25.1 | 746.4 | 771.5 | 3.3 |
| 56 | Oral disorders | 23.2 | 762.3 | 785.5 | 2.9 |
| 57 | Maternal sepsis and other pregnancy related infection | 23.0 | 359.1 | 382.2 | 6.0 |
| 58 | Osteoarthritis | 19.6 | 330.5 | 350.1 | 5.6 |
| 59 | Schizophrenia | 19.1 | 51.2 | 70.3 | 27.1 |
| 60 | Animal contact | 18.5 | 749.0 | 767.5 | 2.4 |
| 61 | Congenital anomalies | 17.5 | 76.2 | 93.7 | 18.7 |
| 62 | Preterm birth complications | 17.1 | 46.8 | 63.9 | 26.7 |
| 63 | Foreign body | 16.7 | 622.1 | 638.8 | 2.6 |
| 64 | Self-harm | 14.9 | 230.8 | 245.8 | 6.1 |
| 65 | Other transport injuries | 14.9 | 255.6 | 270.5 | 5.5 |
| 66 | Cardiomyopathy and myocarditis | 14.6 | 93.7 | 108.3 | 13.4 |
| 67 | Gout | 14.4 | 143.1 | 157.5 | 9.2 |
| 68 | Fire, heat and hot substances | 14.2 | 190.0 | 204.2 | 7.0 |
| 69 | Hemoglobinopathies and hemolytic anemias | 11.7 | 33.4 | 45.1 | 26.0 |
| 70 | Treatment of hyperlipidemia | 11.4 | 120.9 | 132.3 | 8.6 |
| 71 | Colon and rectum cancers | 11.0 | 23.7 | 34.6 | 31.7 |
| 72 | Other neoplasms | 10.6 | 44.4 | 54.9 | 19.2 |
| 73 | Brain and nervous system cancers | 9.7 | 30.6 | 40.3 | 24.0 |
| 74 | Varicella | 8.3 | 99.1 | 107.4 | 7.7 |
| 75 | Otitis media | 6.8 | 534.3 | 541.1 | 1.3 |
| 76 | Sexually transmitted diseases excluding HIV | 5.2 | 229.8 | 235.0 | 2.2 |
| 77 | Tobacco intervention | 4.6 | 166.7 | 171.2 | 2.7 |
| 78 | Peripheral vascular disease | 4.4 | 16.4 | 20.8 | 21.3 |
| 79 | Bladder cancer | 4.3 | 15.6 | 20.0 | 21.7 |
| 80 | Meningitis | 4.0 | 9.9 | 13.9 | 28.8 |
| 81 | Rheumatoid arthritis | 3.2 | 34.6 | 37.8 | 8.5 |
| 82 | Maternal hemorrhage | 2.1 | 167.1 | 169.2 | 1.3 |
| 83 | Other neonatal disorders | 2.0 | 6.2 | 8.2 | 24.5 |
| 84 | Aortic aneurysm | 2.0 | 10.1 | 12.0 | 16.5 |
| 85 | Breast cancer | 1.7 | 91.0 | 92.7 | 1.8 |
| 86 | Hypertensive disorders of pregnancy | 1.5 | 2.7 | 4.2 | 36.0 |
| 87 | Multiple sclerosis | 1.3 | 8.5 | 9.9 | 13.6 |
| 88 | Treatment of obesity | 1.3 | 10.5 | 11.8 | 11.1 |
| 89 | Prostate cancer | 1.2 | 6.4 | 7.7 | 16.2 |
| 90 | Conduct disorder | 1.0 | 19.0 | 20.0 | 5.2 |
| 91 | Hepatitis | 1.0 | 3.6 | 4.6 | 21.8 |
| 92 | Attention-deficit/hyperactivity disorder | 0.9 | 16.6 | 17.5 | 5.3 |
| 93 | Vascular intestinal disorders | 0.9 | 1.0 | 1.9 | 48.4 |
| 94 | Non-Hodgkin lymphoma | 0.7 | 2.0 | 2.7 | 24.8 |
| 95 | Neglected tropical diseases and malaria | 0.6 | 10.5 | 11.1 | 5.0 |
| 96 | Parkinson's disease | 0.5 | 4.5 | 5.0 | 9.6 |
| 97 | HIV/AIDS | 0.5 | 1.3 | 1.8 | 26.3 |
| 98 | Other mental and behavioral disorders | 0.4 | 6.8 | 7.2 | 5.9 |
| 99 | Interstitial lung disease and pulmonary sarcoidosis | 0.4 | 1.5 | 1.9 | 20.9 |
| 100 | Stomach cancer | 0.3 | 1.4 | 1.7 | 15.4 |
| 101 | Leukemia | 0.2 | 1.0 | 1.2 | 20.2 |
| 102 | Pancreatic cancer | 0.2 | 0.6 | 0.8 | 29.1 |
| 103 | Counselling services | 0.2 | 10.0 | 10.2 | 2.2 |
| 104 | Non-melanoma skin cancer | 0.1 | 5.8 | 5.9 | 2.0 |
| 105 | Tension-type headache | 0.1 | 6.9 | 7.0 | 1.5 |
| 106 | Drowning | 0.1 | 2.6 | 2.7 | 3.5 |
| 107 | Collective violence and legal intervention | 0.1 | 5.4 | 5.5 | 1.5 |
| 108 | Liver cancer | 0.1 | 0.5 | 0.5 | 14.6 |
| 109 | Endocarditis | 0.1 | 1.2 | 1.3 | 5.2 |
| 110 | Cervical cancer | 0.1 | 1.0 | 1.0 | 5.2 |
| 111 | Autistic spectrum disorders | 0.0 | 0.7 | 0.8 | 5.7 |
| 112 | Protein-energy malnutrition | 0.0 | 0.2 | 0.2 | 20.2 |
| 113 | Neonatal encephalopathy (birth asphyxia and birth trauma) | 0.0 | 0.1 | 0.1 | 19.5 |
| 114 | Ovarian cancer | 0.0 | 0.2 | 0.2 | 11.4 |
| 115 | Eating disorders | 0.0 | 0.4 | 0.5 | 3.8 |
| 116 | Obstructed labor | 0.0 | 0.0 | 0.0 | 60.3 |
| 117 | Kidney cancer | 0.0 | 0.2 | 0.2 | 7.7 |
| 118 | Esophageal cancer | 0.0 | 0.1 | 0.1 | 8.2 |
| 119 | Other nutritional deficiencies | 0.0 | 0.1 | 0.1 | 9.5 |
| 120 | Family planning | 0.0 | 1.1 | 1.1 | 0.5 |
| 121 | Tuberculosis | 0.0 | 0.0 | 0.1 | 9.7 |
| 122 | Encephalitis | 0.0 | 0.0 | 0.0 | 9.9 |
| 123 | Multiple myeloma | 0.0 | 0.0 | 0.0 | 10.6 |
| 124 | Mouth cancer | 0.0 | 0.0 | 0.0 | 2.4 |
| 125 | Idiopathic intellectual disability | 0.0 | 0.0 | 0.0 | 2.9 |
| 126 | Pneumoconiosis | 0.0 | 0.0 | 0.0 | 1.3 |
| 127 | Hodgkin lymphoma | 0.0 | 0.0 | 0.0 | 1.2 |
| 128 | Testicular cancer | 0.0 | 0.0 | 0.0 | 2.0 |
| 129 | Social services | 0.0 | 0.0 | 0.0 | 0.8 |
| 130 | Uterine cancer | 0.0 | 0.0 | 0.0 | 2.7 |
| 131 | Gallbladder and biliary tract cancer | 0.0 | 0.0 | 0.0 | 1.1 |
| 132 | Exposure to forces of nature | 0.0 | 0.0 | 0.0 | 0.2 |
| 133 | Thyroid cancer | 0.0 | 0.0 | 0.0 | 0.5 |
| 134 | Malignant skin melanoma | 0.0 | 0.0 | 0.0 | 0.3 |
| 135 | Leprosy | 0.0 | 0.0 | 0.0 | 1.8 |
| 136 | Iodine deficiency | 0.0 | 0.0 | 0.0 | 3.9 |
| 137 | Tetanus | 0.0 | 0.0 | 0.0 | 0.2 |
| 138 | Measles | 0.0 | 0.0 | 0.0 | 0.6 |
| 139 | Acute glomerulonephritis | 0.0 | 0.0 | 0.0 | n/a |
| 140 | Diphtheria | 0.0 | 0.0 | 0.0 | n/a |
| 141 | Donor | 0.0 | 0.0 | 0.0 | n/a |
| 142 | Hemolytic disease in fetus and newborn and other neonatal jaundice | 0.0 | 0.0 | 0.0 | n/a |
| 143 | Hypertensive heart disease | 0.0 | 0.0 | 0.0 | n/a |
| 144 | Intestinal infectious diseases | 0.0 | 0.0 | 0.0 | n/a |
| 145 | Nasopharynx cancer | 0.0 | 0.0 | 0.0 | n/a |
| 146 | Other pharynx cancer | 0.0 | 0.0 | 0.0 | n/a |
| 147 | Rheumatic heart disease | 0.0 | 0.0 | 0.0 | n/a |
| 148 | Sepsis and other infectious disorders of the newborn baby | 0.0 | 0.0 | 0.0 | n/a |
| 149 | Vitamin A deficiency | 0.0 | 0.0 | 0.0 | n/a |
| 150 | Whooping cough | 0.0 | 0.0 | 0.0 | n/a |

*Note:* Conditions ranked in terms of total estimated ED spending among the subset of encounters who were hospitalized following their ED visit. Please note that ED spending estimates were mapped to only 150 of the 154 individual DEX health conditions, as four health conditions (larynx cancer, well newborn care, well dental, and well person) either did not appear in the NEDS data or were systematically excluded as a primary diagnosis. Lastly, some estimates for conditions with low levels of ED spending show a non-zero value for percentage of ED spending attributable to the hospitalized cohort of patients (ED spending among hospitalized group by Condition X/Total ED spending by Condition X) due to decimal point limitations of the prior columns (0.0).

## S8 Table. Total ED Spending, Absolute and Relative Changes in ED Spending, and Changes in ED Spending Per Visit, All Conditions

|  |  | ED Spending | | | | | | ED Spending Per Visit | | |
| --- | --- | --- | --- | --- | --- | --- | --- | --- | --- | --- |
|  |  | Total Spending 2016 | | Absolute Change | | Relative Change | | 2006 | 2016 | Relative Change |
|  |  | $ Billions | Rank | $ Billions (2016 - 2006) | Rank | AROC (%) | Rank | $ | $ | AROC (%) |
| # | **All Health Conditions** | **136.61** | **n/a** | **57.38** | **n/a** | **4.4** | **n/a** | **660.05** | **943.16** | **3.4** |
| 1 | Road injuries | 9.88 | 1 | 4.24 | 2 | 4.8 | 45 | 954.28 | 1606.98 | 5.1 |
| 2 | Urinary diseases | 9.41 | 2 | 4.39 | 1 | 5.2 | 44 | 959.73 | 1289.57 | 2.8 |
| 3 | Falls | 8.46 | 3 | 2.87 | 4 | 3.0 | 71 | 613.57 | 856.53 | 3.2 |
| 4 | Other digestive diseases | 5.47 | 4 | 2.30 | 8 | 4.2 | 56 | 809.02 | 1071.44 | 2.6 |
| 5 | Low back and neck pain | 5.18 | 5 | 2.49 | 5 | 5.6 | 39 | 657.95 | 1001.82 | 4.0 |
| 6 | Skin and subcutaneous diseases | 5.01 | 6 | 2.31 | 7 | 5.3 | 42 | 389.83 | 575.99 | 3.7 |
| 7 | Unintentional injuries due to mechanical forces | 4.76 | 7 | 0.79 | 26 | 1.1 | 87 | 472.91 | 701.63 | 3.9 |
| 8 | Ischemic heart disease | 4.59 | 8 | 1.48 | 13 | 1.9 | 84 | 1349.54 | 1714.86 | 2.4 |
| 9 | Inflammatory bowel disease | 4.57 | 9 | 2.37 | 6 | 6.6 | 30 | 798.72 | 1245.93 | 4.3 |
| 10 | Hypertension | 4.44 | 10 | 1.87 | 9 | 3.8 | 58 | 1128.53 | 1238.2 | 0.8 |
| 11 | Gallbladder and biliary diseases | 4.16 | 11 | 3.02 | 3 | 12.4 | 3 | 1029.37 | 1808.6 | 5.7 |
| 12 | Lower respiratory tract infections | 4.12 | 12 | 1.30 | 16 | 2.9 | 73 | 490.36 | 661.7 | 3.0 |
| 13 | Other unintentional injuries | 3.55 | 13 | 1.45 | 14 | 4.5 | 52 | 475.53 | 699.38 | 3.7 |
| 14 | Asthma | 3.52 | 14 | 1.45 | 15 | 4.7 | 47 | 631.61 | 910.39 | 3.6 |
| 15 | Chronic obstructive pulmonary disease | 3.31 | 15 | 1.71 | 10 | 5.2 | 43 | 952.2 | 1286.51 | 3.1 |
| 16 | Other musculoskeletal conditions, including joint pain | 3.29 | 16 | 1.63 | 11 | 5.8 | 36 | 600.79 | 770.85 | 2.3 |
| 17 | Gynecological diseases | 3.16 | 17 | 1.28 | 17 | 4.7 | 49 | 825.62 | 1119.68 | 3.0 |
| 18 | Appendicitis | 3.07 | 18 | 0.68 | 28 | 2.0 | 82 | 1220.23 | 2630.62 | 7.9 |
| 19 | Other neurological conditions | 2.97 | 19 | 1.53 | 12 | 6.2 | 32 | 712.85 | 991.6 | 3.1 |
| 20 | Upper respiratory tract infections | 2.73 | 20 | 0.86 | 23 | 3.4 | 64 | 314.41 | 398.78 | 2.2 |
| 21 | Diabetes mellitus | 2.34 | 21 | 1.07 | 19 | 4.7 | 48 | 739.71 | 929.56 | 2.3 |
| 22 | Interpersonal violence | 2.09 | 22 | 0.67 | 29 | 3.4 | 65 | 708.71 | 1117.61 | 4.5 |
| 23 | Migraine | 1.95 | 23 | 1.03 | 20 | 7.2 | 24 | 591.8 | 1123.03 | 6.5 |
| 24 | Other chronic respiratory diseases | 1.92 | 24 | 1.14 | 18 | 8.3 | 17 | 576.5 | 991.7 | 5.3 |
| 25 | Cerebrovascular disease | 1.72 | 25 | 0.59 | 31 | 2.2 | 80 | 1047.16 | 1395.76 | 3.0 |
| 26 | Anxiety disorders | 1.61 | 26 | 0.94 | 22 | 8.1 | 20 | 563.11 | 820.77 | 3.7 |
| 27 | Indirect maternal complication of a preexisting medical condition | 1.57 | 27 | 0.83 | 25 | 7.0 | 27 | 604.95 | 953.57 | 4.7 |
| 28 | Sense organ diseases | 1.57 | 28 | 0.69 | 27 | 4.7 | 50 | 330.54 | 503.33 | 3.7 |
| 29 | Heart Failure | 1.54 | 29 | 0.02 | 71 | -2.0 | 105 | 1092.89 | 1004.9 | -0.8 |
| 30 | Atrial fibrillation and flutter | 1.45 | 30 | 0.58 | 32 | 2.9 | 72 | 1097.95 | 1293.99 | 1.6 |
| 31 | Cirrhosis of the liver | 1.38 | 31 | 0.96 | 21 | 10.7 | 8 | 926.14 | 1990.03 | 8.1 |
| 32 | Other infectious diseases | 1.19 | 32 | 0.27 | 45 | 2.1 | 81 | 376.62 | 492.23 | 2.5 |
| 33 | Septicemia | 1.13 | 33 | 0.84 | 24 | 12.1 | 5 | 473.16 | 576.17 | 1.9 |
| 34 | Endocrine, metabolic, blood, and immune conditions | 1.04 | 34 | 0.64 | 30 | 8.6 | 15 | 805.04 | 1265.14 | 4.6 |
| 35 | Gastritis and duodenitis | 1.01 | 35 | 0.34 | 39 | 3.2 | 68 | 954.92 | 1216.39 | 2.3 |
| 36 | Dementia | 0.97 | 36 | 0.19 | 50 | 0.4 | 91 | 2535.83 | 2390.57 | -0.5 |
| 37 | Depressive disorders | 0.94 | 37 | 0.30 | 41 | 3.2 | 69 | 632.46 | 854.88 | 3.0 |
| 38 | Other cardiovascular and circulatory diseases | 0.90 | 38 | 0.35 | 38 | 3.6 | 62 | 835.04 | 988.37 | 1.8 |
| 39 | Epilepsy | 0.87 | 39 | 0.42 | 34 | 6.3 | 31 | 898.73 | 1016.95 | 1.2 |
| 40 | Oral disorders | 0.79 | 40 | 0.25 | 47 | 3.2 | 67 | 242.89 | 318.02 | 2.6 |
| 41 | Poisonings | 0.78 | 41 | 0.57 | 33 | 13.2 | 2 | 610.38 | 836.47 | 3.0 |
| 42 | Complications of abortion | 0.77 | 42 | 0.29 | 43 | 4.2 | 55 | 1415.68 | 2071.47 | 3.9 |
| 43 | Animal contact | 0.77 | 43 | 0.28 | 44 | 3.9 | 57 | 373.93 | 597.26 | 4.6 |
| 44 | Diarrheal diseases | 0.75 | 44 | 0.41 | 35 | 7.0 | 26 | 610.7 | 983.87 | 4.6 |
| 45 | Paralytic ileus and intestinal obstruction | 0.66 | 45 | 0.35 | 37 | 5.8 | 37 | 718.66 | 862.87 | 1.9 |
| 46 | Iron-deficiency anemia | 0.65 | 46 | 0.40 | 36 | 8.7 | 14 | 786.82 | 1473.34 | 6.6 |
| 47 | Foreign body | 0.64 | 47 | 0.25 | 46 | 4.3 | 54 | 438.09 | 580.48 | 2.5 |
| 48 | Other maternal disorders | 0.56 | 48 | 0.30 | 42 | 7.2 | 25 | 667.24 | 981.56 | 4.0 |
| 49 | Otitis media | 0.54 | 49 | -0.02 | 147 | -0.4 | 98 | 236.74 | 266.19 | 1.1 |
| 50 | Other substance use disorders | 0.53 | 50 | 0.31 | 40 | 8.3 | 18 | 593.83 | 838.89 | 3.4 |
| 51 | Inguinal or femoral hernia | 0.50 | 51 | 0.25 | 48 | 5.5 | 40 | 940.06 | 1320.06 | 3.3 |
| 52 | Alcohol use disorders | 0.46 | 52 | 0.12 | 56 | 2.5 | 76 | 554.33 | 565.29 | 0.1 |
| 53 | Pregnancy and postpartum care | 0.40 | 53 | 0.18 | 52 | 5.6 | 38 | 534.49 | 703.94 | 2.8 |
| 54 | Pancreatitis | 0.40 | 54 | 0.15 | 55 | 3.8 | 60 | 674.21 | 862.91 | 2.5 |
| 55 | Maternal sepsis and other pregnancy related infection | 0.38 | 55 | 0.20 | 49 | 7.2 | 23 | 643.54 | 1129.17 | 5.8 |
| 56 | Osteoarthritis | 0.35 | 56 | 0.17 | 53 | 5.4 | 41 | 823.01 | 925.66 | 1.1 |
| 57 | Acute renal failure | 0.33 | 57 | 0.19 | 51 | 6.8 | 29 | 467.69 | 673.18 | 3.7 |
| 58 | Other transport injuries | 0.27 | 58 | 0.17 | 54 | 10.0 | 10 | 942.93 | 1271.4 | 2.9 |
| 59 | Bipolar disorder | 0.27 | 59 | 0.08 | 58 | 3.3 | 66 | 548.88 | 731.78 | 2.9 |
| 60 | Self-harm | 0.25 | 60 | -0.12 | 150 | -4.5 | 135 | 836.23 | 1171.93 | 3.3 |
| 61 | Chronic kidney diseases | 0.24 | 61 | 0.10 | 57 | 3.1 | 70 | 1704 | 1759.69 | 0.2 |
| 62 | Sexually transmitted diseases excluding HIV | 0.23 | 62 | 0.00 | 83 | -0.5 | 99 | 867.15 | 678.88 | -2.4 |
| 63 | Fire, heat and hot substances | 0.20 | 63 | 0.03 | 69 | 1.0 | 88 | 365.91 | 504.18 | 3.1 |
| 64 | Tobacco cessation | 0.17 | 64 | 0.08 | 59 | 4.6 | 51 | 876.03 | 840.54 | -0.6 |
| 65 | Maternal hemorrhage | 0.17 | 65 | -0.04 | 149 | -3.4 | 125 | 735.07 | 614.26 | -1.7 |
| 66 | Peptic ulcer disease | 0.17 | 66 | 0.05 | 64 | 2.6 | 75 | 748.84 | 1006.08 | 3.1 |
| 67 | Gout | 0.16 | 67 | 0.07 | 60 | 4.3 | 53 | 417.13 | 600.98 | 3.4 |
| 68 | Hyperlipidemia | 0.13 | 68 | 0.03 | 68 | 0.3 | 94 | 1805.58 | 1441.56 | -2.2 |
| 69 | Trachea, bronchus, and lung cancers | 0.12 | 69 | 0.05 | 62 | 3.8 | 59 | 748.95 | 1393.6 | 6.3 |
| 70 | Cardiomyopathy and myocarditis | 0.11 | 70 | 0.03 | 66 | 2.2 | 79 | 1377.63 | 2187.75 | 4.5 |
| 71 | Varicella | 0.11 | 71 | 0.05 | 65 | 4.8 | 46 | 303.59 | 365.48 | 1.7 |
| 72 | Hypertensive heart disease | 0.10 | 72 | 0.05 | 63 | 6.2 | 33 | 779.97 | 996.79 | 2.5 |
| 73 | Congenital anomalies | 0.09 | 73 | 0.02 | 72 | 2.3 | 77 | 1812.02 | 1809.86 | -0.1 |
| 74 | Breast cancer | 0.09 | 74 | 0.06 | 61 | 9.5 | 12 | 984.19 | 2461.57 | 9.5 |
| 75 | Schizophrenia | 0.07 | 75 | -0.02 | 148 | -2.9 | 118 | 528.51 | 366.37 | -3.7 |
| 76 | Preterm birth complications | 0.06 | 76 | 0.01 | 75 | 2.6 | 74 | 8686.47 | 9591.36 | 1.0 |
| 77 | Other cancers | 0.05 | 77 | 0.03 | 67 | 8.5 | 16 | 825.35 | 1947.08 | 8.9 |
| 78 | Hemoglobinopathies and hemolytic anemias | 0.05 | 78 | 0.00 | 85 | 0.0 | 95 | 642.4 | 567.66 | -1.3 |
| 79 | Brain and nervous system cancers | 0.04 | 79 | 0.02 | 70 | 8.3 | 19 | 801.16 | 1996.38 | 9.6 |
| 80 | Rheumatoid arthritis | 0.04 | 80 | 0.01 | 73 | 3.5 | 63 | 693.28 | 910 | 2.6 |
| 81 | Rheumatic heart disease | 0.04 | 81 | 0.00 | 145 | -2.3 | 111 | 657.18 | 1769.83 | 10.4 |
| 82 | Colon and rectum cancers | 0.03 | 82 | 0.00 | 77 | -1.4 | 103 | 782.68 | 868.85 | 0.5 |
| 83 | Peripheral vascular disease | 0.02 | 83 | 0.00 | 130 | -2.8 | 116 | 1429.38 | 1234.03 | -1.4 |
| 84 | Conduct disorder | 0.02 | 84 | 0.00 | 80 | 2.3 | 78 | 444.06 | 525.64 | 1.7 |
| 85 | Bladder cancer | 0.02 | 85 | 0.01 | 74 | 7.5 | 21 | 759.75 | 1698.9 | 8.2 |
| 86 | Attention-deficit/hyperactivity disorder | 0.02 | 86 | 0.00 | 139 | -0.9 | 101 | 630.51 | 424.98 | -3.9 |
| 87 | Meningitis | 0.01 | 87 | 0.00 | 86 | 1.5 | 85 | 796.57 | 792.77 | -0.2 |
| 88 | Aortic aneurysm | 0.01 | 88 | 0.00 | 140 | -3.5 | 126 | 1575.97 | 1228.84 | -2.5 |
| 89 | Morbid-obesity | 0.01 | 89 | 0.00 | 79 | 2.0 | 83 | 1512.35 | 1764.47 | 1.2 |
| 90 | Neglected tropical diseases and malaria | 0.01 | 90 | 0.00 | 144 | -2.1 | 106 | 442.32 | 497.04 | 1.0 |
| 91 | Counselling services | 0.01 | 91 | 0.00 | 141 | -2.1 | 107 | 727.25 | 596.75 | -2.2 |
| 92 | Multiple sclerosis | 0.01 | 92 | 0.00 | 142 | -2.3 | 110 | 675.45 | 639.09 | -0.7 |
| 93 | Hemolytic disease in fetus and newborn and other neonatal jaundice | 0.01 | 93 | 0.01 | 76 | 12.2 | 4 | 132.27 | 238.15 | 6.1 |
| 94 | Other neonatal disorders | 0.01 | 94 | 0.00 | 82 | 6.9 | 28 | 66.93 | 111.62 | 5.3 |
| 95 | Prostate cancer | 0.01 | 95 | 0.00 | 146 | -7.6 | 149 | 1097.55 | 878.74 | -2.1 |
| 96 | Sepsis and other infectious conditions of the newborn baby | 0.01 | 96 | 0.00 | 78 | 9.8 | 11 | 154.28 | 328.61 | 7.9 |
| 97 | Other mental and behavioral disorders | 0.01 | 97 | 0.00 | 87 | 0.3 | 93 | 677.65 | 646.19 | -0.8 |
| 98 | Tension-type headache | 0.01 | 98 | 0.00 | 88 | 0.8 | 90 | 610.51 | 640.75 | 0.3 |
| 99 | Non-melanoma skin cancer | 0.01 | 99 | 0.00 | 84 | 8.7 | 13 | 525.3 | 1160.93 | 7.7 |
| 100 | Collective violence and legal intervention | 0.01 | 100 | 0.00 | 143 | -3.3 | 124 | 512.87 | 608.29 | 1.5 |
| 101 | Parkinson's disease | 0.01 | 101 | 0.00 | 132 | -4.1 | 133 | 907.77 | 702.22 | -2.6 |
| 102 | Other pharynx cancer | 0.00 | 102 | 0.00 | 81 | 16.1 | 1 | 506.37 | 1512.74 | 11.5 |
| 103 | Hepatitis | 0.00 | 103 | 0.00 | 133 | -2.2 | 108 | 452.41 | 471.45 | 0.4 |
| 104 | Hypertensive conditions of pregnancy | 0.00 | 104 | 0.00 | 129 | -2.7 | 115 | 825.17 | 691.54 | -1.7 |
| 105 | Drowning | 0.00 | 105 | 0.00 | 131 | -2.4 | 112 | 845.99 | 925.87 | 0.7 |
| 106 | Non-Hodgkin lymphoma | 0.00 | 106 | 0.00 | 134 | -4.8 | 139 | 879.68 | 744.04 | -1.6 |
| 107 | Interstitial lung disease and pulmonary sarcoidosis | 0.00 | 107 | 0.00 | 138 | -4.9 | 141 | 890.2 | 785.07 | -1.6 |
| 108 | Vascular intestinal conditions | 0.00 | 108 | 0.00 | 136 | -5.7 | 144 | 815.11 | 781.62 | -1.2 |
| 109 | Whooping cough | 0.00 | 109 | 0.00 | 89 | 11.9 | 6 | 306.58 | 512.05 | 5.2 |
| 110 | HIV/AIDS | 0.00 | 110 | 0.00 | 135 | -3.5 | 129 | 759.4 | 969.17 | 1.9 |
| 111 | Stomach cancer | 0.00 | 111 | 0.00 | 137 | -4.8 | 140 | 670.9 | 554.24 | -2.1 |
| 112 | Nasopharynx cancer | 0.00 | 112 | 0.00 | 90 | 11.7 | 7 | 454.77 | 1206.59 | 10.0 |
| 113 | Endocarditis | 0.00 | 113 | 0.00 | 92 | 3.7 | 61 | 532.73 | 391.52 | -3.1 |
| 114 | Leukemia | 0.00 | 114 | 0.00 | 125 | -2.2 | 109 | 780.8 | 661.61 | -1.8 |
| 115 | Acute glomerulonephritis | 0.00 | 115 | 0.00 | 91 | 6.1 | 34 | 425.01 | 650.79 | 4.4 |
| 116 | Family planning | 0.00 | 116 | 0.00 | 126 | -2.4 | 113 | 667.99 | 532.19 | -2.3 |
| 117 | Cervical cancer | 0.00 | 117 | 0.00 | 127 | -2.9 | 119 | 1063.11 | 1077.55 | -0.1 |
| 118 | Pancreatic cancer | 0.00 | 118 | 0.00 | 128 | -4.5 | 136 | 641.12 | 530.54 | -2.0 |
| 119 | Autistic spectrum disorders | 0.00 | 119 | 0.00 | 93 | 0.9 | 89 | 617.47 | 538.21 | -1.7 |
| 120 | Liver cancer | 0.00 | 120 | 0.00 | 120 | -3.5 | 127 | 625.41 | 490.48 | -2.3 |
| 121 | Eating disorders | 0.00 | 121 | 0.00 | 95 | -0.8 | 100 | 601.07 | 602.61 | 0.0 |
| 122 | Organ donation (harvesting) | 0.00 | 122 | 0.00 | 96 | 0.0 | 96 | 1380.34 | 1390.98 | -1.7 |
| 123 | Kidney cancer | 0.00 | 123 | 0.00 | 119 | -3.7 | 131 | 1056.8 | 1188.27 | 0.9 |
| 124 | Protein-energy malnutrition | 0.00 | 124 | 0.00 | 124 | -6.4 | 146 | 586.42 | 466.79 | -2.5 |
| 125 | Ovarian cancer | 0.00 | 125 | 0.00 | 123 | -4.7 | 138 | 1179.81 | 1310.47 | 1.0 |
| 126 | Neonatal encephalopathy (birth asphyxia and birth trauma) | 0.00 | 126 | 0.00 | 94 | 5.8 | 35 | 248.27 | 295.73 | 1.8 |
| 127 | Esophageal cancer | 0.00 | 127 | 0.00 | 121 | -6.5 | 147 | 957.58 | 845.51 | -0.8 |
| 128 | Other nutritional deficiencies | 0.00 | 128 | 0.00 | 118 | -4.1 | 134 | 908.8 | 661.58 | -2.8 |
| 129 | Tuberculosis | 0.00 | 129 | 0.00 | 117 | -3.0 | 120 | 374.73 | 464.26 | 1.8 |
| 130 | Pneumoconiosis | 0.00 | 130 | 0.00 | 122 | -6.2 | 145 | 775.65 | 587.85 | -2.7 |
| 131 | Hodgkin lymphoma | 0.00 | 131 | 0.00 | 115 | -3.3 | 123 | 1506.84 | 2556.54 | 4.2 |
| 132 | Obstructed labor | 0.00 | 132 | 0.00 | 116 | -3.7 | 132 | 286.41 | 251.73 | -1.5 |
| 133 | Mouth cancer | 0.00 | 133 | 0.00 | 113 | -3.5 | 128 | 1173.91 | 2036.79 | 5.1 |
| 134 | Encephalitis | 0.00 | 134 | 0.00 | 97 | 0.4 | 92 | 1675.07 | 1656.03 | -1.6 |
| 135 | Idiopathic intellectual disability | 0.00 | 135 | 0.00 | 114 | -3.2 | 121 | 1093.76 | 1913 | 4.1 |
| 136 | Social services | 0.00 | 136 | 0.00 | 99 | -0.9 | 102 | 471.08 | 339.24 | -3.2 |
| 137 | Exposure to forces of nature | 0.00 | 137 | 0.00 | 112 | -3.3 | 122 | 2042.33 | 3708.57 | 6.3 |
| 138 | Multiple myeloma | 0.00 | 138 | 0.00 | 111 | -2.8 | 117 | 943.61 | 1145.01 | 1.7 |
| 139 | Testicular cancer | 0.00 | 139 | 0.00 | 110 | -2.6 | 114 | 1298.16 | 1347.23 | -0.2 |
| 140 | Vitamin A deficiency | 0.00 | 140 | 0.00 | 98 | 10.5 | 9 | 1784.8 | 839.85 | -7.2 |
| 141 | Uterine cancer | 0.00 | 141 | 0.00 | 100 | 0.0 | 97 | 817.29 | 813.48 | -0.6 |
| 142 | Diphtheria | 0.00 | 142 | 0.00 | 102 | -1.7 | 104 | n/a | n/a | n/a |
| 143 | Malignant skin melanoma | 0.00 | 143 | 0.00 | 109 | -5.0 | 142 | 1864.91 | 2607.53 | 4.7 |
| 144 | Thyroid cancer | 0.00 | 144 | 0.00 | 106 | -3.7 | 130 | 2701.11 | 3073.22 | 2.6 |
| 145 | Gallbladder and biliary tract cancer | 0.00 | 145 | 0.00 | 108 | -4.6 | 137 | 846.96 | 1710.16 | 7.4 |
| 146 | Leprosy | 0.00 | 146 | 0.00 | 107 | -12.9 | 150 | 2402.99 | 1756.73 | -3.0 |
| 147 | Tetanus | 0.00 | 147 | 0.00 | 105 | -7.2 | 148 | n/a | n/a | n/a |
| 148 | Intestinal infectious diseases | 0.00 | 148 | 0.00 | 101 | 7.3 | 22 | n/a | n/a | n/a |
| 149 | Iodine deficiency | 0.00 | 149 | 0.00 | 104 | -5.2 | 143 | 97.38 | 65.89 | -3.8 |
| 150 | Measles | 0.00 | 150 | 0.00 | 103 | 1.3 | 86 | 230.61 | 198.75 | -1.6 |

*Note:* ED spending estimates were mapped to only 150 of the 154 individual DEX health conditions, as four health conditions (larynx cancer, well newborn care, well dental, and well person) either did not appear in the NEDS data or were systematically excluded as a primary diagnosis. Absolute change = the difference in total ED spending between 2016 and 2006. The corresponding rank summarizes how health conditions compared to one another in terms of absolute changes in spending. The relative change measure is a population-standardized Annualized Rate of Change (AROC), which accounts for changes in the population between 2016 and 2006 (i.e., assumes the same population in 2016 as 2006). It provides a relative estimate of the change in spending growth (or spending per visit growth) noted from 2006 and 2016 for all conditions, and then by each individual health condition. All estimates are reported in 2016 USD ($).

# **Appendix References**

1. Dieleman JL, Cao J, Chapin A, Chen C, Li Z, Liu A, et al. US Health Care Spending by Payer and Health Condition, 1996-2016. JAMA. 2020;323: 863–884. doi:10.1001/jama.2020.0734

2. Kliff S. Surprise Medical Bills, the High Cost of Emergency Department Care, and the Effects on Patients. JAMA Intern Med. 2019;179: 1457–1458. doi:10.1001/jamainternmed.2019.3448

3. Healthcare Cost and Utilization Project (HCUP). Nationwide Emergency Department Sample (NEDS), 2016. 2016 [cited 18 Nov 2019]. Available: https://www.hcup-us.ahrq.gov/db/nation/neds/NEDS_Introduction_2016.jsp

4. Centers for Medicare & Medicaid Services. National Health Expenditure Data. [cited 1 Feb 2020]. Available: https://www.cms.gov/Research-Statistics-Data-and-Systems/Statistics-Trends-and-Reports/NationalHealthExpendData

5. Medical Expenditure Panel Survey. Household Component Summary Data Tables. In: Emergency Room Services-Median and Mean Expenses per Person With Expense and Distribution of Expenses by Source of Payment: United States, 2014 [Internet]. [cited 1 Feb 2020]. Available: https://meps.ahrq.gov/mepsweb/data_stats/download_data_files.jsp

6. Agency for Healthcare Research and Quality. Medical Expenditure Panel Survey. [cited 1 Feb 2020]. Available: https://www.meps.ahrq.gov/mepsweb/

7. Dieleman JL, Baral R, Birger M, Bui AL, Bulchis A, Chapin A, et al. US Spending on Personal Health Care and Public Health, 1996-2013. JAMA. 2016;316: 2627–2646. doi:10.1001/jama.2016.16885

8. Galarraga JE, Pines JM. Costs of ED episodes of care in the United States. The American Journal of Emergency Medicine. 2016;34: 357–365. doi:10.1016/j.ajem.2015.06.001
